# A Trifunctional Ni–P/Fe–P Collaborated Electrocatalyst Enables Self‐Powered Energy Systems

**DOI:** 10.1002/advs.202201594

**Published:** 2022-05-22

**Authors:** Rui Yang, Xiaozhong Zheng, Minkai Qin, Binbin Lin, Xiaoyun Shi, Yong Wang

**Affiliations:** ^1^ Advanced Materials and Catalysis Group State Key Laboratory of Clean Energy Utilization Center of Chemistry for Frontier Technologies Institute of Catalysis Department of Chemistry Zhejiang University Hangzhou 310028 P. R. China; ^2^ College of Chemistry and Molecular Engineering Zhengzhou University Zhengzhou 450001 P. R. China

**Keywords:** electrocatalyst, energy systems, hydrogen generation, self‐powered

## Abstract

Recently, extensive research efforts have been devoted to drive electrocatalytic water‐splitting for hydrogen generation by electricity or solar cells. However, electricity from power grid and the intermittent property of sunlight inevitably brings about environmental pollution and energy loss. Thus, a novelty energy system for simultaneous generating H_2_ from solar energy and overcoming the intermittence of sunlight is highly desirable. Herein, a self‐powered energy system with solar energy as the sole input energy is successfully assembled by integrated Zn–air batteries with stable output voltage, solar cells, and water splitting electrolyzer to efficient H_2_ production. Specially, the Zn–air batteries are charged by the solar cell to store intermitted solar energy as electricity during light reaction. Under unassisted light reaction, the batteries could release electric energy to drive H_2_ production. Therefore, the aim for simultaneous generating H_2_ and eliminating the restrictions of intermittent sunlight are realized. The solar‐to‐hydrogen efficiency and solar‐to‐water splitting device efficiency of the self‐powered energy system are up to 4.6% and 5.9%, respectively. This work provides the novel design systems for H_2_ production and the usage of renewable energy.

## Graphical abstract

Based on the multifunctional Ni–P/Fe–P collaborated electrocatalyst, the self‐powered energy system is assembled to generate H_2_ fully powered from solar energy, achieving the energy and substances circulation. Meanwhile, the solar‐to‐hydrogen efficiency and solar‐to‐water splitting device efficiency of this system are up to 4.6% and 5.9%, respectively.

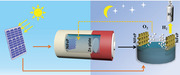

## 1 Introduction

The energy crisis and environmental issues derived from the usage of fossil fuel direct the attention to sustainable energy sources and renewable energy technologies. Hydrogen, as a clean energy with the advantages of high energy density and wide sources, offers an alternative for traditional fossil fuel.^[^ ^1^, ^2^ ^]^ Among the various hydrogen energy techniques, electrochemical hydrogen evolution reaction (HER) from water‐splitting is regarded as a promising approach to H_2_ production because of the mild reaction conditions, high‐purity products, and extensive resources. Significant progress has been developed to electrocatalytic water‐splitting directly driven by electricity or solar cells.^[^ ^3^, ^4^, ^5^ ^]^ The former method has to use electricity from power grid, which is energy‐consuming and uneconomical. The latter of the intermittence of sunlight usually requires connections of energy storage devices, which suffer from complicated structures and external energy loss. Therefore, it is highly desirable to design and develop novel energy systems for H_2_ generation, which is expected to fully powered by solar energy with the storage of solar energy to overcome the intermittence of sunlight.

Zn–air batteries with oxygen reduction reactions (ORR) and oxygen evolution reactions (OER) electrochemical processes are undertaken intensive attention, which can function not only as feasible energy storage, like solar energy, but also as conversion technologies with high discharge voltage.^[^ ^6^, ^7^ ^]^ Generally, due to the overpotential of HER and OER, the demand voltage of water splitting is at 1.5–2.3 V, higher than the theory thermodynamic potential of 1.23 V.^[^ ^8^, ^9^ ^]^ The two Zn–air batteries in series could effectively drive overall water splitting. Also, the Zn–air battery could play an important role to store solar energy in light reaction and release energy into electricity in dark reaction to drive water splitting, so that the intermittent sunlight was fully utilized and the sunlight restrictions were eliminated.

The trifunctional stable electrocatalyst for HER, OER, and ORR is vital for building the above novel systems. To date, the state‐of‐the‐art electrocatalysts are still precious metals and their oxides, like IrO_2_, RuO_2_, and Pt/C. But their high costs and scarcity hamper the practical application in multiply functional systems.^[^ ^10^, ^11^, ^12^ ^]^ Therefore, developing the alternative electrocatalyst with earth‐abundant elements, as well as trifunction toward HER/OER/ORR is urgently required. Recently, transition metals (e.g., Fe, Co, Ni)^[^ ^13^, ^14^, ^15^ ^]^ and their derivatives (e.g., phosphides,^[^ ^16^, ^17^ ^]^ carbides,^[^ ^18^, ^19^ ^]^ sulfides,^[^ ^20^, ^21^ ^]^ etc.) have received great attention for their cost‐effective, abundant reserves, and easy modification, exhibiting promising alternatives to precious metals. Among above various electrocatalytic materials, the metal and phosphorus sites of the transition‐metals phosphides sites played the role of proton/hydride‐acceptor centers with moderate bonding energy of reaction intermediate in OER.^[^ ^22^, ^23^ ^]^ Additionally, according to reported works, iron phosphide materials exhibited excellent ORR performance due to activated O_2_ molecules by Fe—P bond.^[^ ^24^, ^25^ ^]^ During HER process, when there existed P and Fe/Ni, the P atom with strong electronegativity attracted electron from Ni/Fe atoms and then the negative P atom could capture protons. Therefore, P element combined with Fe/Ni could adjust the binding energy between metal and active H atoms, which is essential for HER performance.^[^ ^26^, ^27^, ^28^ ^]^ Furthermore, compared with single metal, binary metal phosphides^[^ ^29^, ^30^ ^]^ showed better catalytic performance, benefiting from the tuned coordination condition and electronic structure after coupling secondary metal. Besides the electronic environment, the hydrophilicity, which was influenced by morphology, also affects the electrocatalytic activity.^[^ ^31^, ^32^ ^]^ Thus, there are huge potentials for binary metal phosphides towards multifunctional HER/OER/ORR.

In this study, we developed a Ni–P/Fe–P collaborated trifunctional electrocatalyst NiFeP with hedgehog‐like nanoarrays morphology, exhibiting robust electrocatalytic activity towards HER/OER/ORR. Based on the Ni–P/Fe–P collaborated electrocatalyst, we proposed and successfully assembled self‐powered energy system with the solar‐to‐hydrogen efficiency and solar‐to‐water splitting device efficiency of 4.6% and 5.9%, respectively. In this system, the H_2_ production was fully powered from solar energy, achieving the energy and substances circulation. Such a novelty energy system with trifunctional electrocatalyst supplies a promising approach for the development of renewable energy devices.

## 2 Results and Discussion

### 2.1 Schematic of a Self‐Powered Energy System

**Scheme 1:**
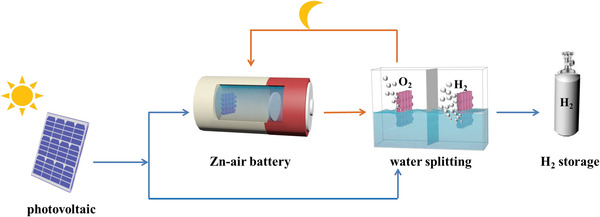
The overall concept of a self‐powered energy system.

A schematic diagram of the self‐powered energy system is shown in **Scheme** **1**. The system consists of silica‐solar cells, Zn–air batteries, and water splitting devices. During light reaction, Zn–air batteries were charged by the solar cell with storing intermitted solar energy as electricity. Meanwhile, the electricity from solar cell could split the water to produce H_2_ fuel. During unassisted light reaction, the charged Zn–air batteries released electric energy to drive water splitting with H_2_ production. Therefore, the water splitting with H_2_ production was driven by solar energy without external nonsolar energy supply. In addition, the produced oxygen from water splitting could supply to Zn–air batteries as cathode active components. Thus, the energy and substances circulation in the self‐powered energy systems were achieved.

### 2.2 Structure Characterizations

**Figure 1:**
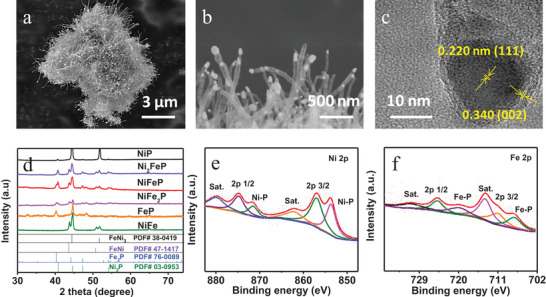
Morphological and structural characterizations of as‐synthesized NiFeP catalysts. a,b) SEM images, c) HRTEM image, d) the XRD pattern of as‐prepared materials, and e,f) High‐resolution XPS spectra of Ni and Fe, respectively.

To realize such a self‐powered energy system, a trifunctional electrocatalyst towards HER/OER/ORR is required. We prepared NiFeP by using oxalic acid as a structure‐directing agent (see experimental section for details). The structures of as‐prepared material were characterized by X‐ray powder diffraction (XRD) (**Figure** **1**d). As shown in Figure 1d, the apparent Ni_2_P (PDF #03‐0953) phase and Fe_2_P (PDF #51‐0943) phase were observed, demonstrating the coexisted Ni_2_P and Fe_2_P phases in NiFeP material.^[^ ^33^ ^]^ The scanning electron microscopy (SEM) images of as‐synthesized electrocatalyst showed hedgehog‐like nanoarrays morphology, which was assembled with bamboo‐like nanotubes (Figure 1a,b, and Figure S1, Supporting Information). The high‐resolution transmission electron microscopy (HRTEM) image (Figure 1c) exhibited the distinct lattice fringes with interplanar distances of 0.340 nm and 0.220 nm, corresponding to the (0 0 2) plane of graphitic carbon and (1 1 1) plane of Fe_2_P/Ni_2_P, respectively.^[^ ^34^, ^35^ ^]^ The selected area electron diffraction (SAED) was conducted in Figure S2 (Supporting Information), from which the clearly diffraction rings were obtained. Among these rings, the marked predominant two rings attributed to the (111) and (300) of Ni_2_P and Fe_2_P, demonstrating the presence of Ni_2_P and Fe_2_P phases in NiFeP material, which was well consistent with XRD pattern and HRTEM image. Thus, bamboo‐like nanotubes were composed of out layer of carbon with 3 nm thick and inlayer particles approximately to 20 nm. The high‐resolution mapping was further conducted in Figure S3 (Supporting Information). As shown, the Fe, Ni, and P were dispersed uniformly in inner particle. The SEM images of contrastive samples were shown in Figure S4 (Supporting Information), which exemplified the exiguous nanotube between graphene sheet of FeP and nanotube twist together of NiP, suggesting that the interaction of Fe and Ni may facilitate the formation of nanoarrays. This kind of structure may benefit for contacting with water and liberating generated gas and therefore accelerate electrocatalytic reaction.^[^ ^32^ ^]^ The surface wettability obtained from the contact angle measurements (Figure S5, Supporting Information) demonstrated the speculation. The NiFeP displayed the smaller contact angle of 117° compared with that of NiFe (144°), FeP (143°), and NiP (131°), exhibiting the favorable contact between electrolyte and electrode surface after the formation of phosphate and coexistence of Ni–P/Fe–P, which further suggested the exaggerated electrocatalytic activity of NiFeP. The enhancement of surface wettability of NiFeP agreed with hedgehog‐like nanoarrays morphology.

To gain more chemical insight toward the prepared materials, the X‐ray photoelectron spectroscopy (XPS), Raman, and the Brunauer‐Emmett‐Teller (BET) were administered to further disclose the surface valence and structure characteristics. The XPS of P 2p spectrum was revealed in Figure S6 (Supporting Information). The apparent peak at 133.8 eV was assigned to P—C bond, proclaiming that the P element successfully doped into carbon skeleton. The appeared P—O bond (134.7 eV) attributed to the surface phosphorus oxidized as a consequence of exposure to air. The metal—P bond presented at 128.9 eV corroborated the existence of metal phosphides. The Ni 2p spectrum (Figure 1e) showed Ni 2p_1/2_ (874.9 eV) and Ni 2p_3/2_ (857 eV) as well as their satellites (880 and 862.3 eV).^[^ ^36^ ^]^ The sharp peaks were observed at 871.5 and 853.7 eV, which were well matched with Ni—P bond. The similar results were observed in Fe 2p spectrum (Figure 1f), in which the five peaks were described into Fe 2p_1/2_ (724.8 eV), Fe 2p_3/2_ (711.1 eV), their satellites (731.0 and 714 eV), and Fe—P bond (720 and 707.3 eV).^[^ ^37^ ^]^ The predominant metal—P bonds substantiated the Ni—P/Fe—P coexisted in synthesized NiFeP materials, which was consistent with XRD pattern. The peaks of N 1s spectrum (Figure S7, Supporting Information) were fitted to oxidized N (402.5 eV), graphitic N (401 eV), pyrrole N (400.3 eV), Fe/Ni‐N (399.4 eV), and pyridinic N (398.1 eV),^[^ ^38^ ^]^ suggesting various bonding mode. The N_2_ adsoprtion–desorption isotherms (Figure S8, Supporting Information) confirmed the abundant mesoporous characteristic and the specific surface of 118 m^2^ g^‐1^, the large surface may be attributed to the hedgehog‐like nanoarrays morphology. The Raman of prepared NiFeP material (Figure S9, Supporting Information) exhibited the highest D/G peak intensity ratio (*I*_D_/*I*_G_ = 0.98). The D bands and G bands were corresponding to disordered carbon and the E_2g_ vibration of the sp^2^‐hybridized graphitic carbon, thus the increased *I*_D_/*I*_G_ indicated the higher degree of defects. According to the reported works,^[^ ^39^, ^40^ ^]^ the defective conductive carbon supported to improve the conductivity, which is reflected in higher current density for HER/OER/ORR. Meanwhile, edge defects can expose more active sites, which are responsible for actually catalytic sites. Thus, the richer defects suggested higher electrocatalytic performance, which was in accordance with SEM of bamboo‐like nanotubes.^[^ ^41^ ^]^ The above characterizations manifest that the as‐prepared NiFeP material is defect‐enriched hedgehog‐like nanoarrays with co‐doped Ni—P and Fe—P groups.

### 2.3 Electrochemical Performance

**Figure 2:**
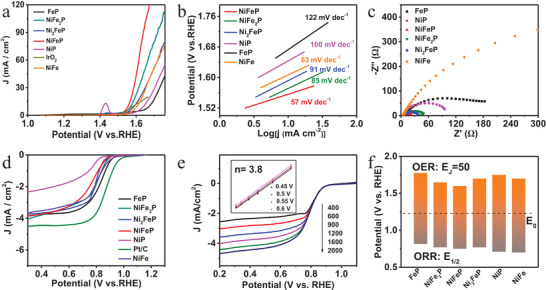
Electrocatalytic performance. a) OER polarization curves, b) corresponding Tafel slopes, c) EIS, and d) ORR polarization curves of as‐prepared materials. e) ORR polarization curves of NiFeP at different rotating speeds and the inset is the K–L plots including the number of electron transfer (*n*). f) Potential differences between the potential of OER at 50 mA cm^−2^ and the half‐wave potential of ORR.

The electrocatalytic activity was carried in the typical three‐electrode system. The OER performance of various Ni/Fe ratios was displayed in **Figure** **2**a. NiFeP exhibited higher activity with the lowest overpotential of 370 mV at 50 mA cm^−2^. While FeP, NiFe_2_P, Ni_2_FeP, and NiP obtained overpotential of 552, 418, 470, and 520 mV, respectively, which showed the superiority of cooperation with Ni–P/Fe–P as well as the importance of the Ni/Fe ratio. Besides, NiFe gained 470 mV at 50 mA cm^−2^ much higher than that of NiFeP implying the strong synergistic effect after phosphorization. As shown in Figure 2b, the Tafel slope of NiFeP was fitted to 57 mV dec^−1^ lower than contrastive samples, implying the more favorable OER reaction kinetics in Ni–P/Fe–P cooperated NiFeP catalyst. Meanwhile, OH* + OH^−^ → O* + H_2_O + e^−^was the rate‐limiting step.^[^ ^42^, ^43^ ^]^ The electrochemical impedance spectroscopy (EIS) analysis and corresponding equivalent circuit of NiFeP material were depicted in Figure 2c and Figure S10 (Supporting Information), respectively. NiFeP exhibited the smallest Nyquist semicircle diameter (*R*_ct_), indicating the fast electron transfer rate during OER process. The potential‐time curves at 10 mA cm^−2^ (Figure S11, Supporting Information) showed well stability of NiFeP material after 35 h. Therefore, the co‐incorporation of Ni–P/Fe–P in NiFeP and the unique hedgehog‐like morphology facilitated the OER performance. Subsequently, the LSV polarization curves for ORR in O_2_‐saturated 0.1 m KOH solution were observed in Figure 2d, in which the onset potential of NiFeP was delivered 0.923 V. The electron transfer numbers were obtained from K–L plots under different rotating speeds (Figure 2e and Figure S12, Supporting Information). As shown, the electron transfer number was 3.8 of fabricated NiFeP, disclosing the four‐electron dominated transfer pathway. Also, the stability of ORR was performed with chronopotentiometry, exhibiting considerable stability compared with Pt/C (Figure S13, Supporting Information).

To further understand the intrinsic activity of NiFeP, the electrochemical surface area (ECSA) was estimated from double‐layer capacitance (*C*_dl_) (Figure S14, Supporting Information). The *C*_dl_ of NiFeP (9.89 mF cm^−2^) was greater than those of FeP (9.57 mF cm^−2^), NiP (8.57 mF cm^−2^), and NiFe (1.63 mF cm^−2^), delivering the highly exposed metal active sites of NiFeP. The gap between the potential of OER at 50 mA cm^−2^ and the half‐wave potential of ORR was shown in Figure 2f. It is precisely apparent that the fabricated NiFeP presented the smallest gap, further elucidating the superior of coexistence of Ni–P/Fe–P. The activity gap is significant for Zn–air batteries.^[^ ^44^ ^]^ The Δ*E* value of *E*_onset_ and *E*_1/2_ potential (Figure S15, Supporting Information) exhibited smaller value, which is a crucial parameter in designing a metal‐air battery. Therefore, the decent catalytical performance of NiFeP may be principally attributed to hedgehog‐like nanoarrays morphology and optimized composition of Ni–P/Fe–P in NiFeP.

To verify the usability of NiFeP in self‐powered energy systems, the HER and water splitting were further explored (Figure S16, Supporting Information). The polarization curves of HER (Figure S16a, Supporting Information) displayed the lower onset potential of NiFeP than NiP and FeP. Also, the stability of NiFeP material towards HER showed in Figure S17 (Supporting Information). The negligible attenuation demonstrated the good stability. Figure S16b (Supporting Information) exhibited the polarization curves of water splitting with lower overpotential of NiFeP than that of the mixture of Pt/C and IrO_2_, describing the superiority of Ni–P/Fe–P cooperation. The gas volume ratio of H_2_/O_2_ displayed 2:1 which was in accord with the theoretical value from water splitting at current density of 25 mA cm^−2^ (Figure S16c, Supporting Information). The continuous bubbles can be seen from the digital of water splitting with NiFeP as cathode and anode (Figure S16d, Supporting Information), exhibiting the practicability of NiFeP with HER and water splitting.

Further, a series of density functional theory (DFT) calculations were conducted to supply mechanistic insight for electrochemcial process. For the HER process, the two typical reaction pathways are (1) H_2_O + e^−^ → H* + OH^−^; (2) H_2_O + e^−^ + H* → H_2_ + OH^−^; (3) H* + H* → H_2_. It is apparent that the absorbed free energy of hydrogen (Δ*G*_H*_) on the catalyst surface is the main parameter to determine the activity.^[^ ^2^, ^45^ ^]^ The one whose Δ*G*_H*_ value approached zero, represented the outstanding performance. Thus, the H adsorption free energy on the surface of NiFeP, NiP, and FeP was calculated. The views of the NiP (111), FeP (111), and NiFeP (111) models of HER were displayed in Figures S18a,b, and S19a (Supporting Information), respectively. The Δ*G*_H*_ value exhibited the 0.04, ‐0.27, and 0.9 eV for NiFeP, NiP, and FeP, respectively (Figure S19b, Supporting Information). Therefore, the NiFeP material possessed best HER performance. When the strong electronegativity of P atom combined with Ni/Fe, it induced charge redistribution and the adjustment of the binding energy between metal and active H atoms, demonstrating the advantage of Ni–P/Fe–P.

According to reported works,^[^ ^46^, ^47^, ^48^ ^]^ the NiFeP material exhibited the in situ formation of thin layer of active NiFeOOH along with phosphates on NiFeP during OER. The lone pair of electrons of phosphorus in 3p orbitals and vacant 3d orbitals could accommodate electrons on surface and enrich local charge density. Therefore, the phosphates can form more active state to boost the OER kinetics. The views of the NiP (111), FeP (111), and NiFeP (111) models of ORR were displayed in Figures S20–S22 (Supporting Information), respectively. Figure S23 (Supporting Information) displayed the reaction pathway of ORR with Gibbs free energy at *U* = 0 (* was the adsorption site on catalyst surfaces). From the free energy diagram, the third step is the rate‐determining step for all materials and the lower free energy of NiFeP would result in a decent theoretical overpotential and better catalytic activity, which was in accordance with experimental results.^[^ ^49^ ^]^

**Figure 3:**
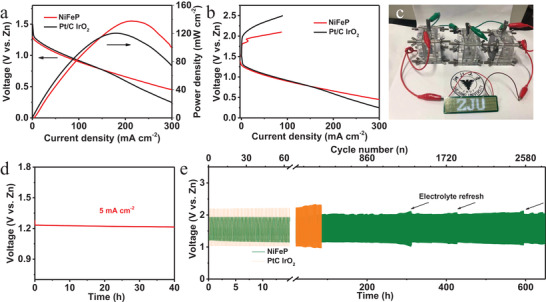
The performance of Zn–air batteries. a) Charge and discharge polarization curves and b) polarization and power density curves of Zn batteries with prepared NiFeP and the mixture of Pt/C and IrO_2_ as cathode, respectively. c) The photo of a LED screen powered by Zn–air batteries. d) Galvanostatic discharge at 5 mA cm^−2^ with NiFeP as cathode. e) Galvanostatic charge‐discharge cycle curves of Zn batteries with NiFeP and the mixture of Pt/C and IrO_2_ at 10 mA cm^−2^.

The Zn–air batteries played an important role in the proposed self‐powered energy systems. Thus, the multifunctional NiFeP was applied in Zn–air batteries to further evaluate its practical utility. The catalyst and Zn plate were regarded as cathode and anode in the assembled batteries, respectively. The mixture of Pt/C and IrO_2_ was also integrated into batteries as comparison. **Figure** **3**a revealed the maximum power density of the Zn–air battery was 138 mW cm^−2^ using NiFeP as cathode, exceeding the 120 mW cm^−2^ of the mixture of Pt/C and IrO_2_ counterpart. The open‐circuit voltage (OCV) was determined to be 1.38 V, which remained stable after long time (Figure S24, Supporting Information), manifesting superior activity. The charge and discharge polarization curves (Figure 3b) for Zn–air battery illustrated the much smaller voltage gap of charge and discharge with NiFeP as cathode compared to that with the mixture of Pt/C and IrO_2_ counterpart, suggesting the better energy utilization efficiency. In addition, a light emitting diode (LED) screen (LED, 3.7 V) (Figure 3c) and pocket fan (Figure S25, Supporting Information) were powered by Zn–air batteries with an excellent operation stability. The stability of assembled batteries was evaluated with long‐term galvanostatic discharge in Figure 3d. The galvanostatic discharge delivered a higher voltage of 1.23 V at the current density of 5 mA cm^−2^ with the negligible performance fading after 40h, exhibiting excellent durability. Furthermore, galvanostatic charge‐discharge cycle curves of the Zn–air battery (Figure 3e) based on NiFeP air‐cathode at current density of 10 mA cm^−2^ exhibited charge voltage of 1.93 V and discharge voltage of 1.21 V, with the voltage gap of 0.72 V and energy efficiency of 63% at the first cycle number. After 1200 cycles (300 h), the voltage gap slightly increased with 0.29 V and energy efficiency decreased to 49%, delivering a slight performance loss. Whereas, the Zn–air batteries with the mixture of Pt/C and IrO_2_ as cathode showed 2.20 and 1.03 V of charge voltage and discharge voltage, respectively, with energy efficiency of 46% in the beginning. After cycling for 85 h, the charge voltage and discharge voltage sharply changed to 2.32 and 0.95 V, respectively, further corroborating outstanding cycling stability of the Zn–air batteries with NiFeP as cathode under highly oxidative operating conditions. The obvious declined activity of the mixture of Pt/C and IrO_2_ resulted from carbon oxidization and the particle agglomeration.^[^ ^50^, ^51^ ^]^ The slightly decayed performance of Zn–air batteries with NiFeP was attributed to carbon oxidization with positive potential during charge. In the meantime, during long‐term cycles, insoluble carbonate salts in electrolyte were produced from CO_2_ in air, resulting in the decreasing of the concentration of electrolyte and hindering gas channel. It can be demonstrated in Figure 3e, the voltage gap can be recovered by a refreshing electrolyte. The above activity proclaimed the strong stability and reversibility of the Zn–air battery on NiFeP air‐cathode, suggesting the well stable of self‐powered energy systems.

**Figure 4:**
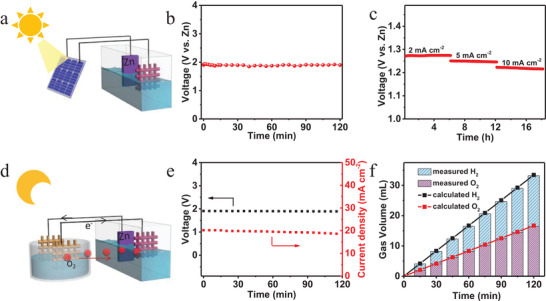
A schematic representation of the self‐driven energy system. a) The schematic of the charge of Zn–air battery by silicon solar cell under light reaction. b) Charging curve of Zn‐air battery powered by silicon solar cell. c) The galvanostatic discharging curves at 2, 5, and 10 mA cm^−2^ after charging. d) The schematic of Zn–air battery driven water splitting under unassisted light reaction. e) The obtained voltage and current density of water splitting and f) the corresponding measured and calculated gas volumes of H_2_ and O_2_.

Based on the multifunctional electrocatalyst NiFeP, a self‐driven energy conversion and storage system was assembled, as illustrated in **Figure** **4**. We conducted the *I*–*V* test of the solar cell (Figure S26, Supporting Information) to demonstrate the driving voltage and current under AM 1.5 illumination. The schematic diagram of the self‐driven energy system under light reaction was presented in Figure 4a, during which the solar cell converted the solar energy to electricity and charged the battery. Figure 4b exhibited the charge performance, in which Zn–air battery was powered by the silicon solar cell exhibiting the steady charge voltage during 120 min. Meanwhile, it can be observed that after charging with solar cell, the stable discharging plateaus kept (Figure 4c) at 1.27, 1.24, and 1.22 V, as the discharge current density increased from 2, 5, to 10 mA cm^−2^, confirming the feasibility of the solar‐charged Zn–air battery. We changed the light intensity to charge the battery in Figure S27 (Supporting Information), suggesting the charge voltage of battery was tightly dependent on light intensity. The batteries stored intermitted solar energy during light reaction and released electric energy to drive H_2_ production under unassisted light reaction (Figure 4d). The digital of two Zn–air batteries in series combined with water splitting showed in Figure S28 (Supporting Information), from which the O_2_ and H_2_ bubbles could be seen clearly in electrode surface. Figure 4e presented that voltage and current density of water splitting maintained at 1.9 V and 20 mA cm^−2^, respectively, which was powered by the Zn–air battery in 120 min. The value of voltage and current density were well consistent with electrocatalyst overall water splitting (Figure S16b, Supporting Information), indicating the practicability of Zn–air driven water splitting. The corresponding measured volumes of H_2_ and O_2_ were depicted in Figure 4f, which matched the theoretical values perfectly for overall water splitting. Concurrently, the faradaic efficiency approached 100% of H_2_ (details in Supporting Information), validating the successful operation of water splitting powered by the Zn–air battery. The solar‐to‐hydrogen conversion efficiency and solar‐to‐water splitting device were up to 4.6% and 5.9%, respectively (details in Supporting Information), which were superior to most recently reported works (Table S1, Supporting Information). The O_2_ produced by water splitting also can transport to Zn–air battery as cathode active components to support discharge reaction (Figure 4d). Besides, the solar cell also could split water directly when sunlight is available. During the process, the voltage and current density of water splitting maintained at 2.04 V and 37.5 mA cm^−2^, respectively. The obtained O_2_ and H_2_ volume were shown in Figure S29a (Supporting Information) and the clearly bubbles were displayed in Figure S29b (Supporting Information), from which the solar‐to‐hydrogen conversion efficiency was calculated to be 7.6%. These results demonstrated the successful construction of a self‐driven energy conversion and storage system, in which the H_2_ production was fully powered from solar energy and the restrictions of intermittent sunlight were eliminated. This system fully utilized solar energy with generation renewable electricity and clean H_2_ fuel without external non‐solar energy supply and by‐product of environment pollution, realizing the energy and substances circulation, which supplies a new approach for energy conversion, storage, and manufacture of clean and renewable energy.

## 3 Conclusion

In summary, the self‐powered energy system was successfully established by integrating Zn–air batteries, silicon solar cells, and water splitting devices, based on the trifunctional Ni–P/Fe–P collaborated NiFeP electrocatalyst with hedgehog‐like nanoarrays morphology. In this system, the batteries were charged by the solar cell with storing intermitted the solar energy as electricity during light reaction. While under unassisted light reaction, the batteries released electric energy to drive H_2_ production. Benefited from the trifunctional NiFeP electrocatalyst, in this system, the conversion efficiency of solar‐to‐hydrogen and solar‐to‐water splitting device were up to 4.6% and 5.9%, respectively. What's more, the oxygen from water splitting can supply to Zn–air batteries as cathode active components. Therefore, this system realized the energy and substances circulation with storage of intermitted solar energy and production of clean H_2_ fuels without external non‐solar energy supply and by‐product of environment pollution. This work supplies a new opportunity for energy conversion and storage, encouraging further research for more practical energy systems.

## 4 Experimental Section

### Chemicals and Reagents

Carbon paper (HCP 120, He Sen), melamine were purchased from Aladdin reagents. Ni(NO_3_)_2_·6H_2_O, Fe(NO_3_)_3_·9H_2_O, NaH_2_PO_2_, and oxalic acid were purchased from Sinopharm Chemical Reagent Co., Ltd. All the reagents were purchased and used without further purification.

### Synthesis of NiFeP

In a typical experiment, 0.363 g (1.25 mmol) Ni(NO_3_)_2_·6H_2_O and 0.5 g (1.25 mmol) Fe(NO_3_)_3_·9H_2_O were dissolved in 10 mL DI water and then 1.26 g melamine was added into another 40 mL water containing 1.58 g (12.5 mmol) oxalic acid. After mixing above solutions, the resulting suspension mixture was refluxed at 80 °C for 6 h and washed by DI water three times after cooled to room temperature. The filter cake was dried by vacuum drying, heated to 800 °C for 2 h in Ar, and further leached with acid in 2.0 m H_2_SO_4_ at 80 °C for 6 h. The obtained black powder was further heated to 350 °C for 2 h in Ar with 2 g NaH_2_PO_2_. P source was put in the front of the powder, the obtained material was named NiFeP. For the comparison, the NiP was fabricated through the similar procedures to that of NiFeP in the absence of Fe^3+^ and with 0.726 g Ni(NO_3_)_2_·6H_2_O. Also, FeP was fabricated in the absence of Ni^2+^ and with 1 g Fe(NO_3_)_3_·9H_2_O. NiFe was synthesized without further phosphorization. 0.239 g (0.825 mmol) Ni(NO_3_)_2_·6H_2_O and 0.676 g (1.675 mmol) Fe(NO_3_)_3_·9H_2_O were used to synthesize NiFe_2_P, meanwhile, 0.487 g (1.675 mmol) Ni(NO_3_)_2_·6H_2_O and 0.333 g (0.825 mmol) Fe(NO_3_)_3_·9H_2_O were used for Ni_2_FeP.

### Characterization

The morphology of materials was characterized with scanning electron microscopy (SEM, JSM‐7600F) and transmission electron microscopy (TEM). X‐ray powder diffraction (XRD) was obtained with a D/max 2500VL/PC diffractometer (Japan) with a Cu K*α* radiation (*λ* = 1.54 Å). X‐ray photon spectroscopy (XPS) was recorded on a Thermo Scientific Escalab 250 Xi with an Al K*α* X‐ray source at 1486.6 eV. N_2_ adsorption/desorption isotherms were measured at 77K (a Micromeritics ASAP 2020 instrument).

### Electrochemical Measurement

All electrochemical measurements (OER, OER, and HER) were evaluated in a typical three‐electrode system (CHI 760E) consisting of a Hg/Hg_2_Cl_2_ reference electrode and a carbon rod counter electrode. All the potentials were standardized to the value with reference to a reversible hydrogen electrode (RHE), according to the equation *E*_RHE_ =$E_{\mathrm{Hg}/Hg_{2}Cl_{2}}$+0.244+0.059*pH. To prepare the catalyst ink, 10 mg catalyst was dispersed in 900 µL ethanol and 100 µL nafion (5%) with sonicing 30 min. 10 µL ink was dropped on RDE or RRDE with 5 mm diameters. 1 m KOH solution was used as electrolyte for HER and OER. While for the ORR experiment, it was tested in O_2_‐saturated 0.1 m KOH and rotated at speeds from 400 to 2000 rpm. The electron transfer number (*n*) was obtained by Koutecky–Levich equation, as previously reported. EIS was tested at 1.53 V versus RHE.

### For Zn–Air Batteries

The batteries consisted of carbon paper, the polished Zn plate, and 6 m KOH electrolyte containing 0.2 m Zn(CH_3_COO)_2_ as cathode, anode, and electrolyte, respectively. IrO_2_‐Pt/C ink was mixed with a mass ratio of 1:1. The charge and discharge polarization curves were conducted by CHI 760E electrochemical workstation. The galvanostatic charge‐discharge curves and cycle curves were recorded by Land system. The charge‐discharge cycle was recorded with 5 min charge and 5 min discharge.

### For the Water Splitting

The test was conducted in two‐electrode system with 1 m KOH as electrolyte. The catalyst ink was dropped in carbon paper (2 cm^2^) as both cathode and anode. The gas production was tested by GC (Fu Li). For the self‐driven water splitting, two Zn–air batteries were used in series.

## Conflict of Interest

The authors declare no conflict of interest.

## Author Contributions

The manuscript was written with contributions of all authors. All authors have given approval to the final version of the manuscript. R.Y. was responsible for the synthesis, electrochemical test, and the manuscript edition. X. Z., M.Q., B.L., and X.S. analyzed the data and revised the paper. Y.W. conducted and supervised this project.

## Supporting information

Supporting Information

Supporting Information

## Data Availability

Research data are not shared.
