# Supplementary material for: A Trifunctional Ni–P/Fe–P Collaborated Electrocatalyst Enables Self‐Powered Energy Systems
Source: Adv Sci (Weinh). 2022 May 22;9(22):2201594. doi: 10.1002/advs.202201594 (PMC9353458; doi:10.1002/advs.202201594)
Supplement: Supplementary file 1 — Supporting Information [file ADVS-9-2201594-s002.pdf]

ADVANCED SCIENCE
Open Access

Supporting Information

for *Adv. Sci.*, DOI 10.1002/advs.202201594

A Trifunctional Ni–P/Fe–P Collaborated Electrocatalyst Enables Self-Powered Energy Systems

*Rui Yang, Xiaozhong Zheng, Minkai Qin, Binbin Lin, Xiaoyun Shi and Yong Wang**

((Supporting Information can be included here using this template))

Supporting Information

A trifunctional Ni-P/Fe-P collaborated electrocatalyst enables self-powered energy systems

*Rui Yang, Xiaozhong Zheng, Minkai Qin, Binbin Lin, Xiaoyun Shi, Yong Wang**

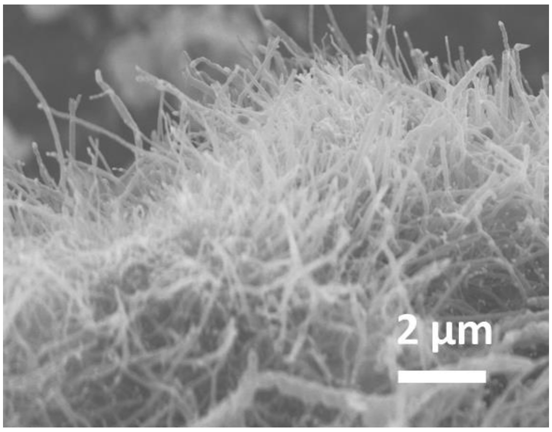

Figure S1. The SEM image of as-synthesized NiFeP materials.

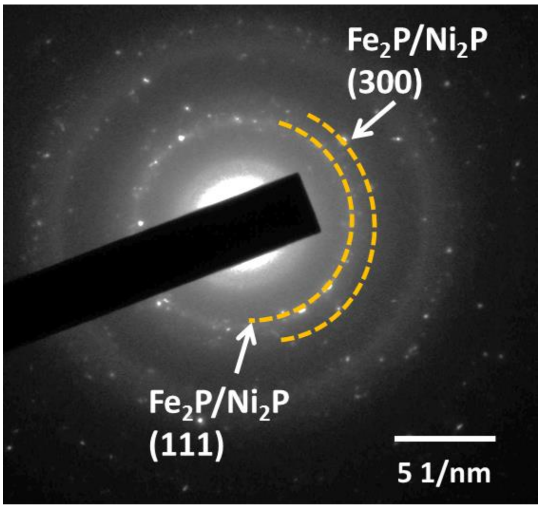

Figure S2. The selected area electron diffraction (SAED) of the obtained NiFeP material.

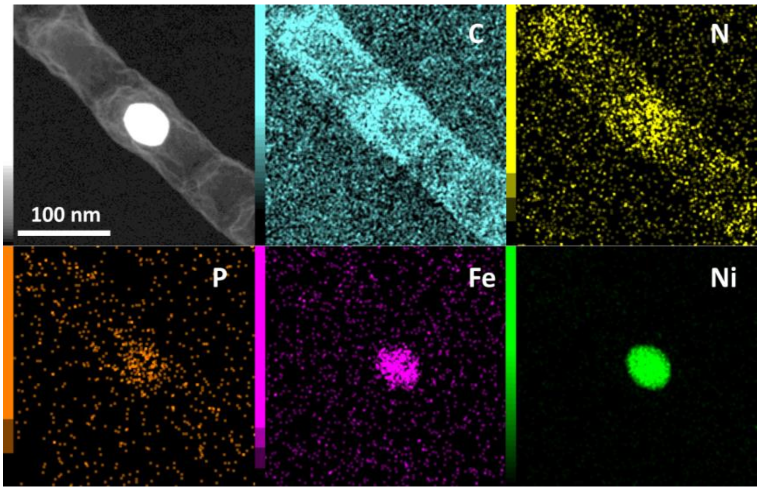

Figure S3. The high-resolution mapping of FeNiP material.

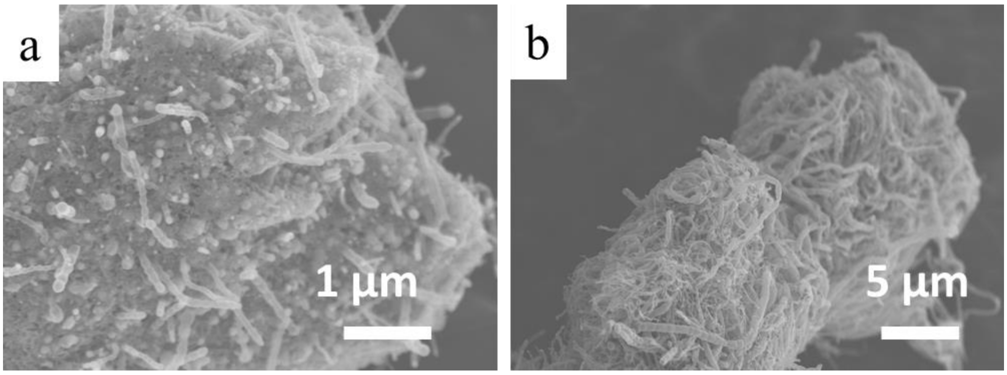

Figure S4. SEM images of (a) FeP and (b) NiP.

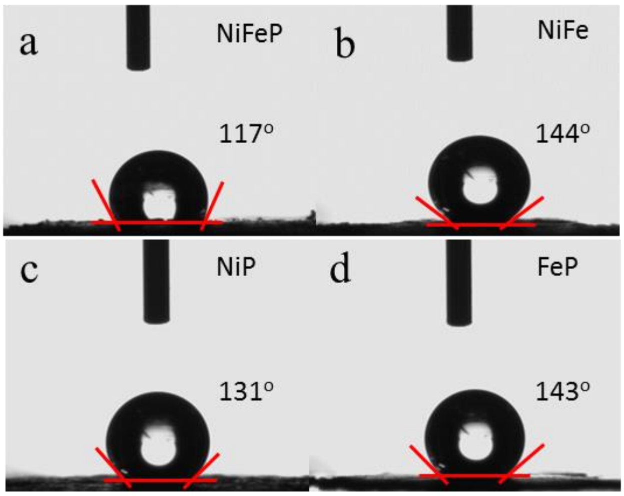

Figure S5. The contact angle measurements of as synthesized materials.(a) NiFeP, (b) NiFe, (c) NiP, and (d) FeP.

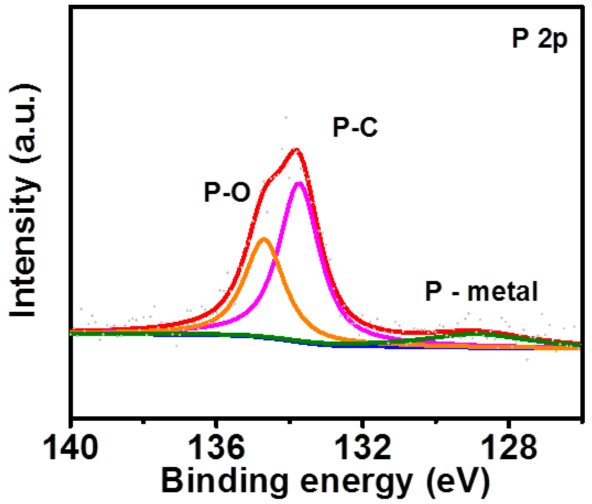

Figure S6. The X-ray photoelectron spectroscopy (XPS) spectra of P element.

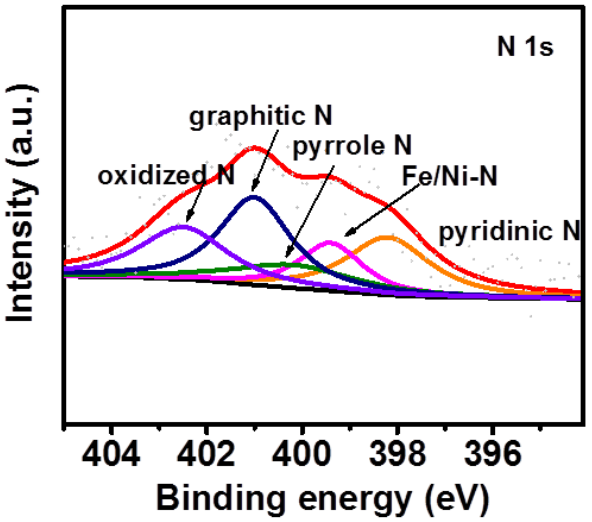

Figure S7. The XPS spectra of N 1s.

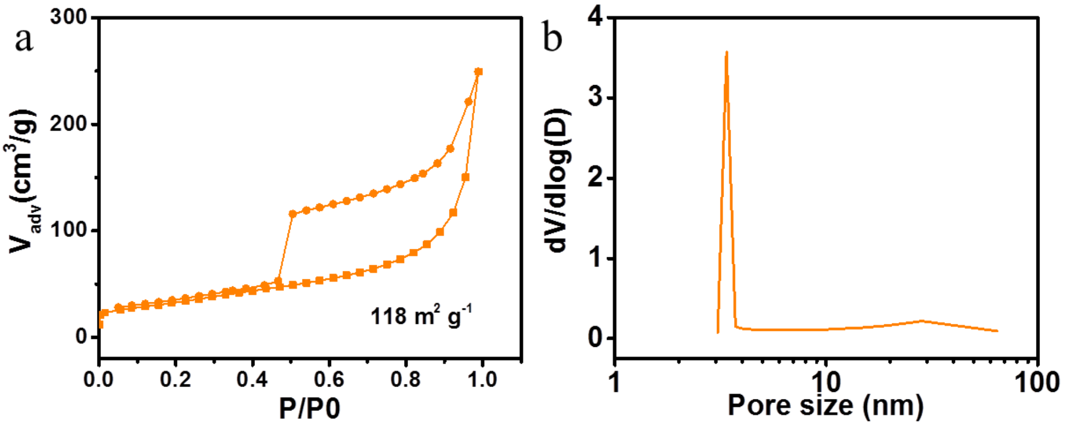

Figure S8. (a) N_2 adsorption-desorption isotherms and (b) pore size distribution curves of NiFeP.

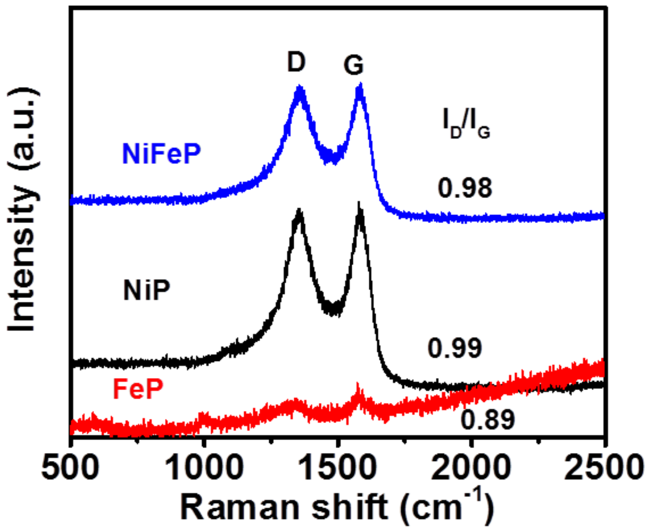

Figure S9. The Raman pattern of NiFeP, NiP and FeP.

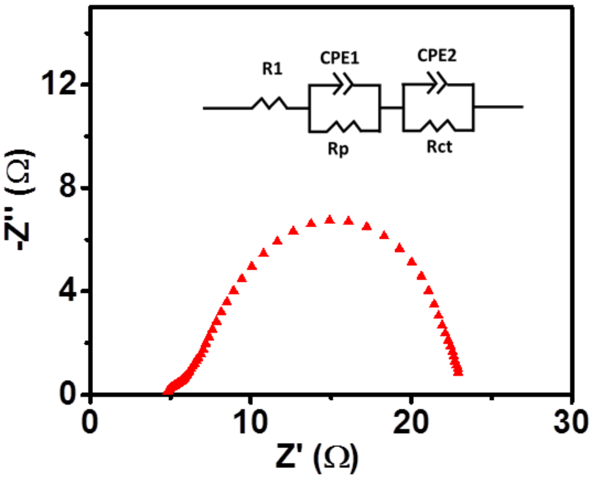

Figure S10. The EIS equivalent circuit of NiFeP material.

The charge transfer resistance (Rct) of NiFeP was 17.37 Ω [1, 2].

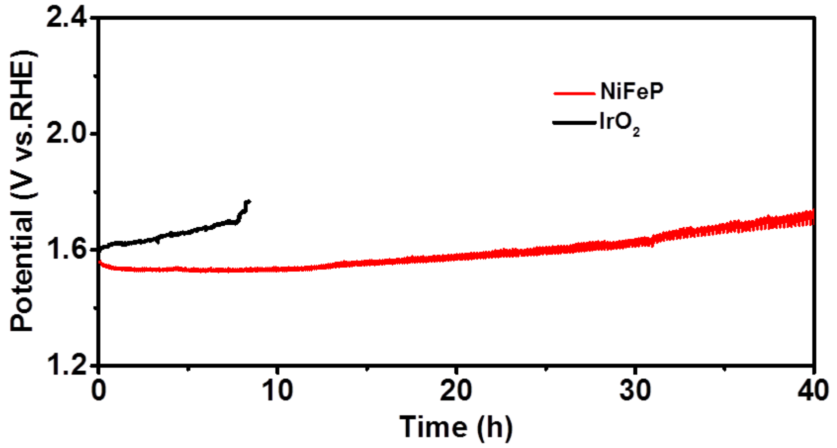

Figure S11. The OER potential–time curves of NiFeP and IrO_2 at 10 mA cm^{-2}.

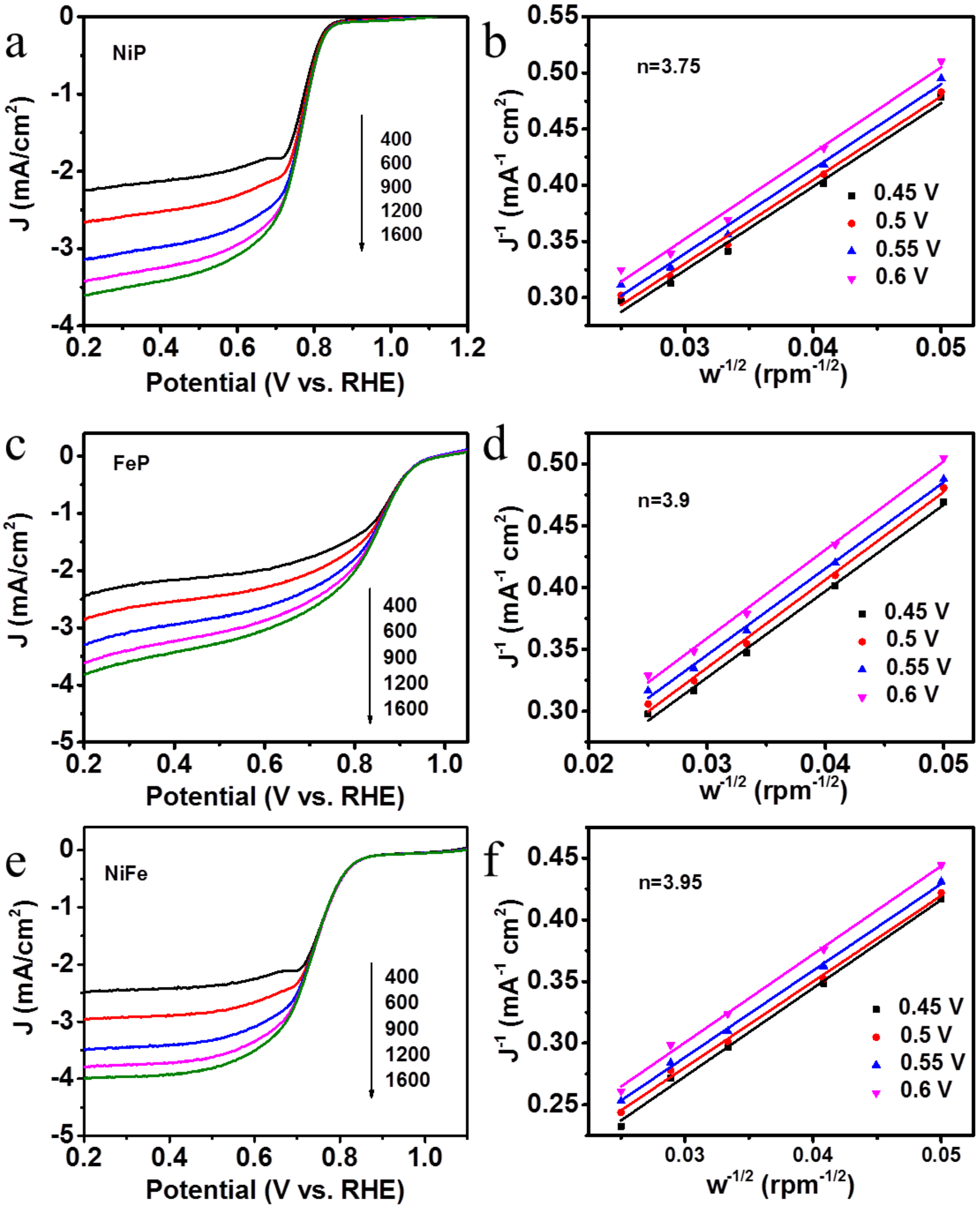

Figure S12. ORR polarization curves of (a) NiP (c) FeP (e) NiFe at different rotating speeds and the K-L plots of (b) NiP (d) FeP (f) NiFe.

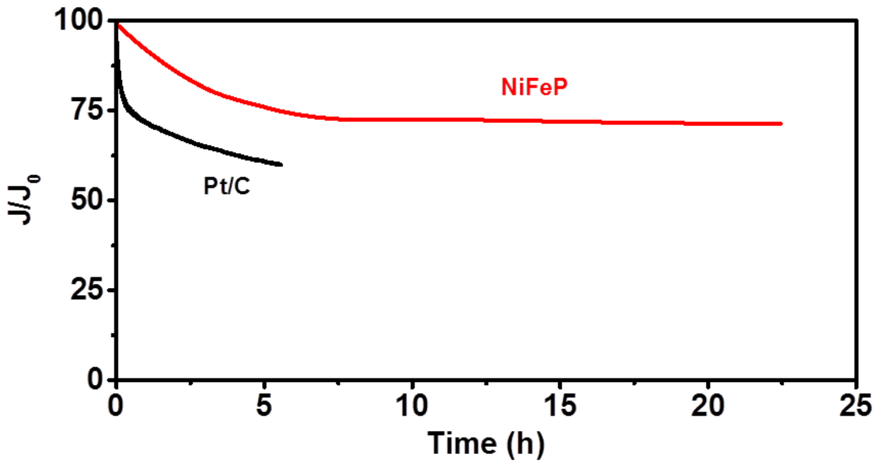

Figure S13. The ORR current–time curve of NiFeP and Pt/C at half-wave potential.

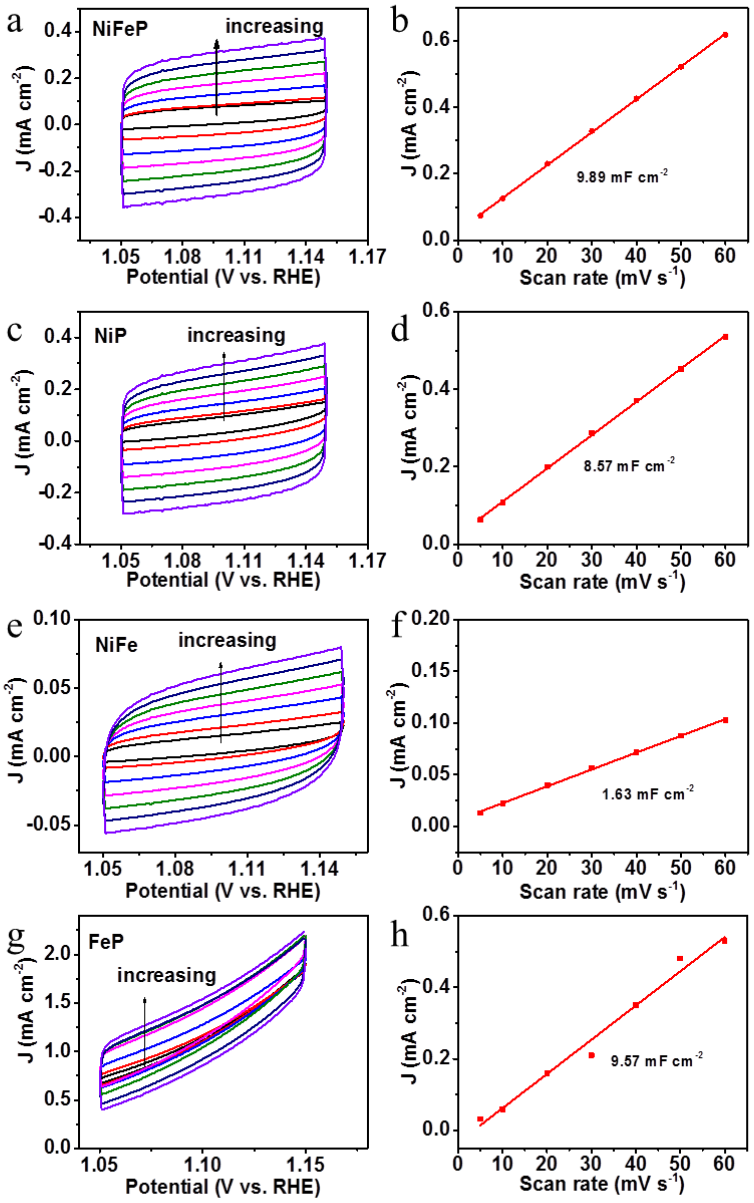

Figure S14. CV curves of (a) NiFeP, (c) NiP, (e) NiFe, and (g) FeP at various scan rates (5, 10, 20, 30, 40, 50, and 60 mV s^{-1}). The current density differences plotted against scan rates for (b) NiFeP, (d) NiP, (f) NiFe, and (h) FeP.

ECSA can be calculated by the following equation:

$$\text{ECSA} = \frac{C_{dl}}{C_S} = \frac{mF\ cm^{-2}}{40\ \mu F\ cm^{-2}\ per\ cm^2_{ECSA} * mass_{cat}} = cm^2_{ECSA} g^{-1}_{cat}$$

$$\text{ECSA}_{(\text{NiFeP})} = \frac{9.89\ \text{mF cm}^{-2}}{40*10^{-3}\ \text{mF cm}^{-2}*1\text{cm}^2*0.1\text{mg}} = 2472\ \text{cm}^2\ \text{mg}^{-1}$$

Cs : the specific capacitance of a flat standard electrode (surface area: 1 cm^2). Usually 20~60 μF cm^{-2}. We take the averaged value of 40 μF cm^{-2}.

Mass loading: 0.1 mg

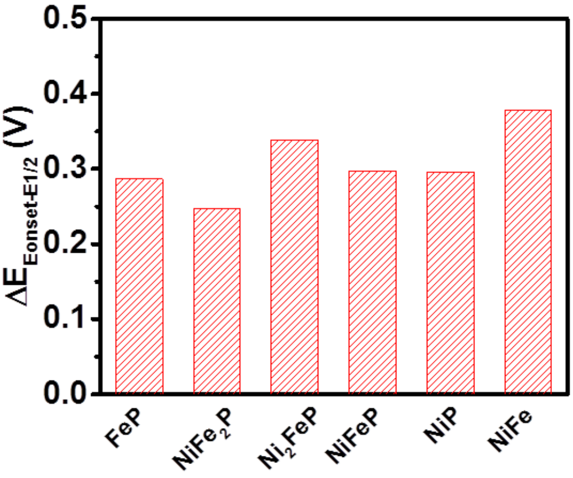

Figure S15. The ΔE value of E_{onset} and $E_{1/2}$ potential.

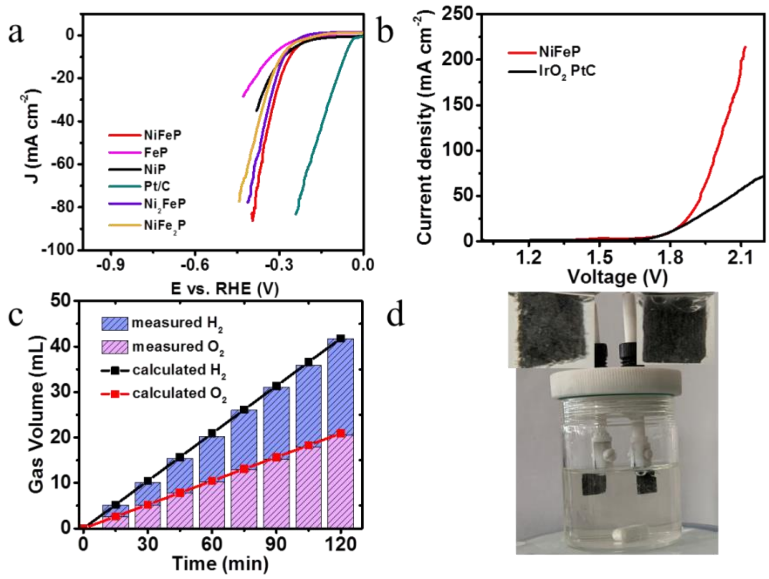

Figure S16. (a) The LSV curves of HER. (b) The polarization curves of water splitting with NiFeP and reference material. (c) The corresponding measured gas volume of H_2 and O_2 and calculated gas volume of H_2 and O_2 of water splitting with NiFeP at 25 mA cm^{-2}. (d) A representative photograph of water splitting with NiFeP as cathode and anode, depicting the H_2 (inset left) and O_2 (inset right) generation during overall water electrolysis.

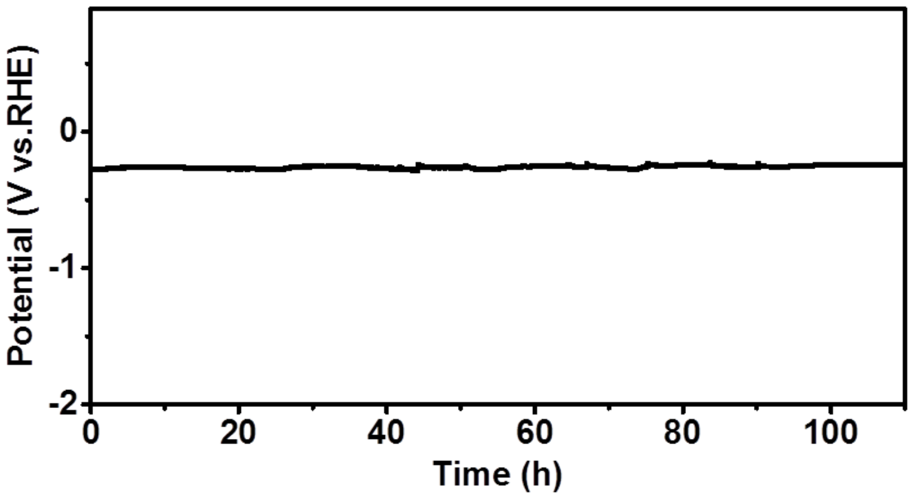

Figure S17. The stability of NiFeP material towards HER at 10 mA cm^{-2} .

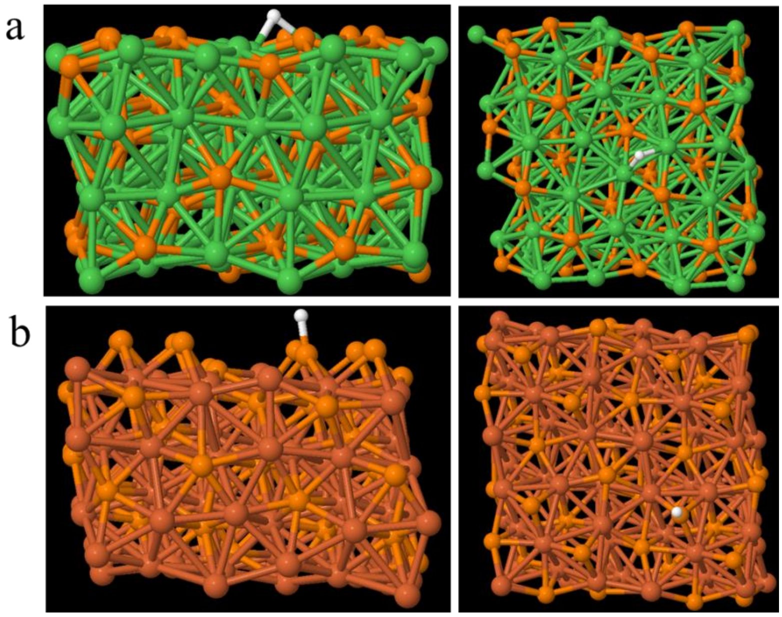

Figure S18. The top and side views of optimized (a) NiP (111) and (b) FeP (111) of HER.

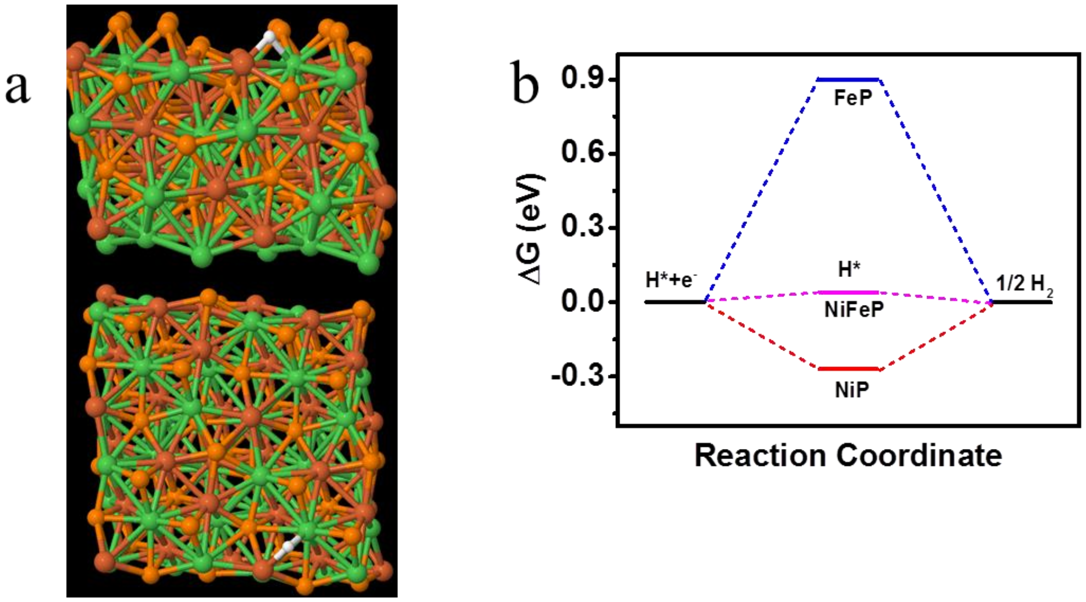

Figure S19. The top and side views of optimized (a) NiFeP (111) and (b) calculated ΔG values for H adsorption on different sites of NiFeP (111), FeP (111), and NiP (111).

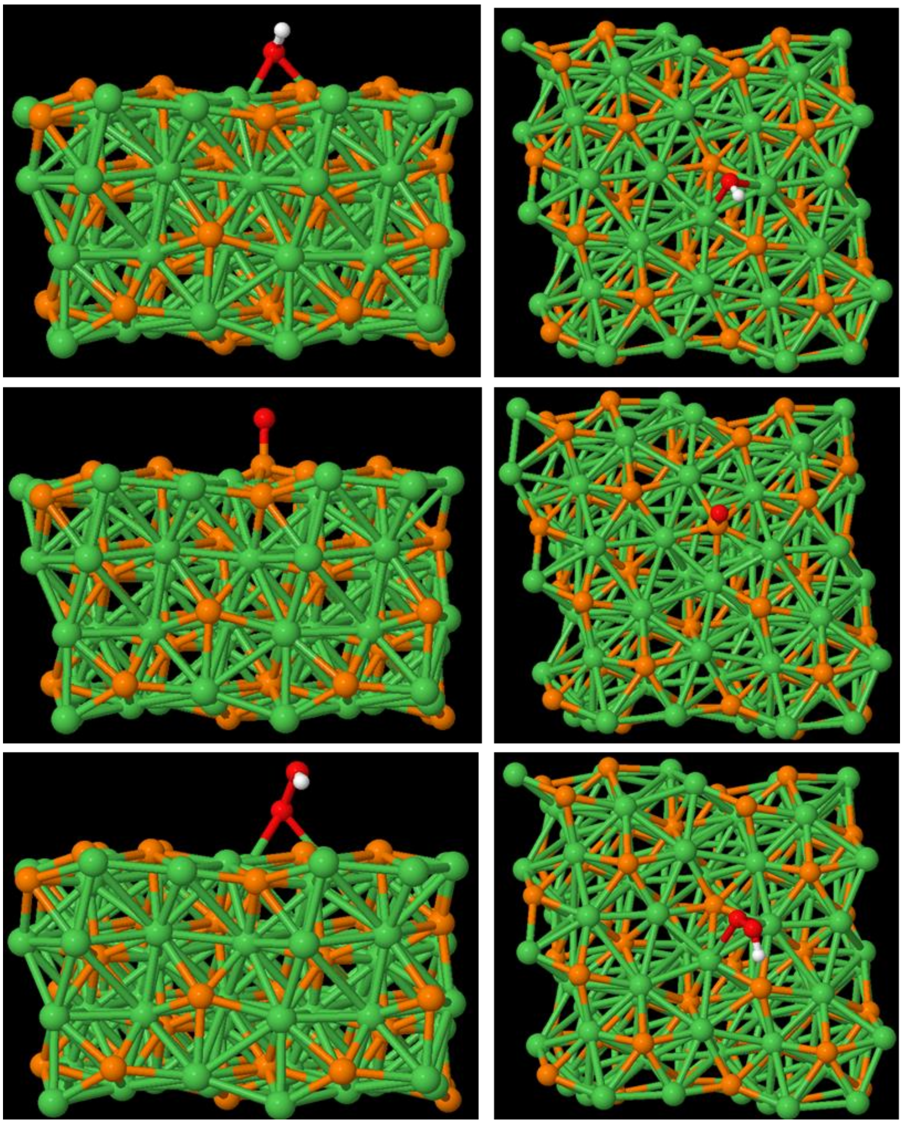

Figure S20. The top and side views of optimized NiP (111) of ORR.

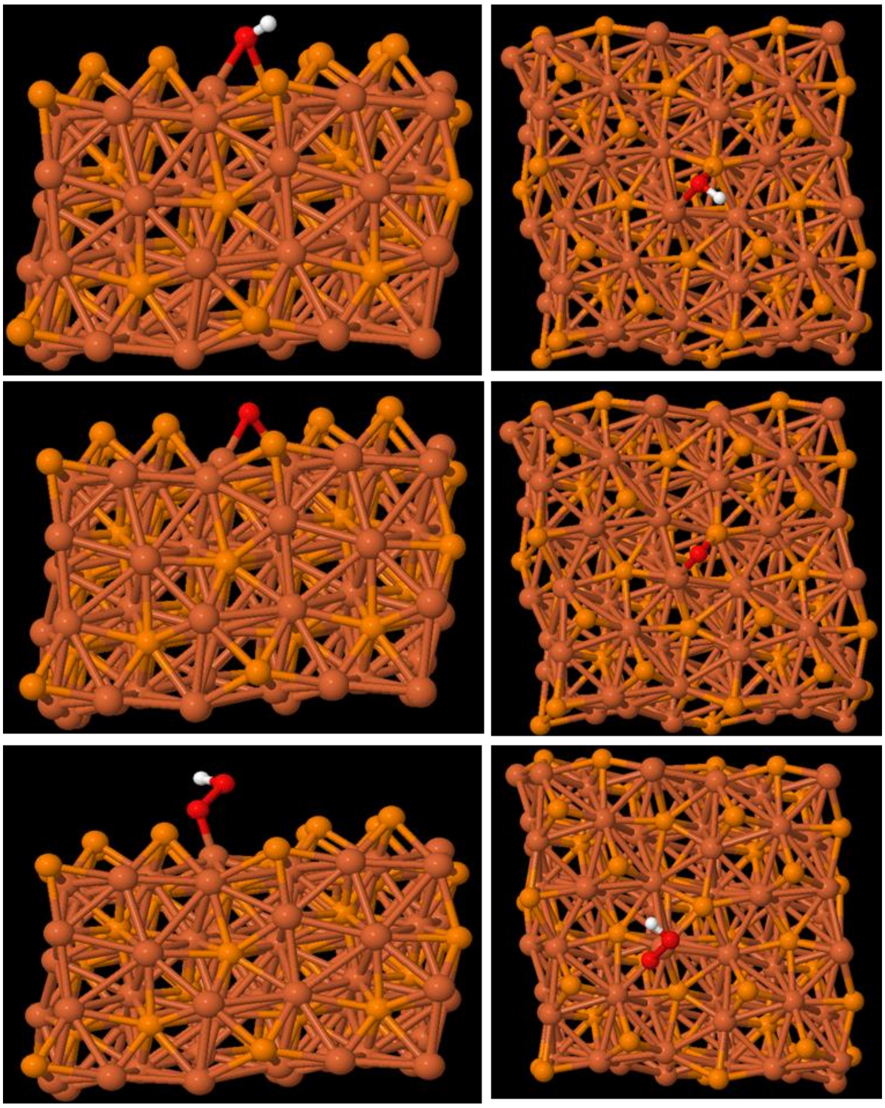

Figure S21. The top and side views of optimized FeP (111) of ORR.

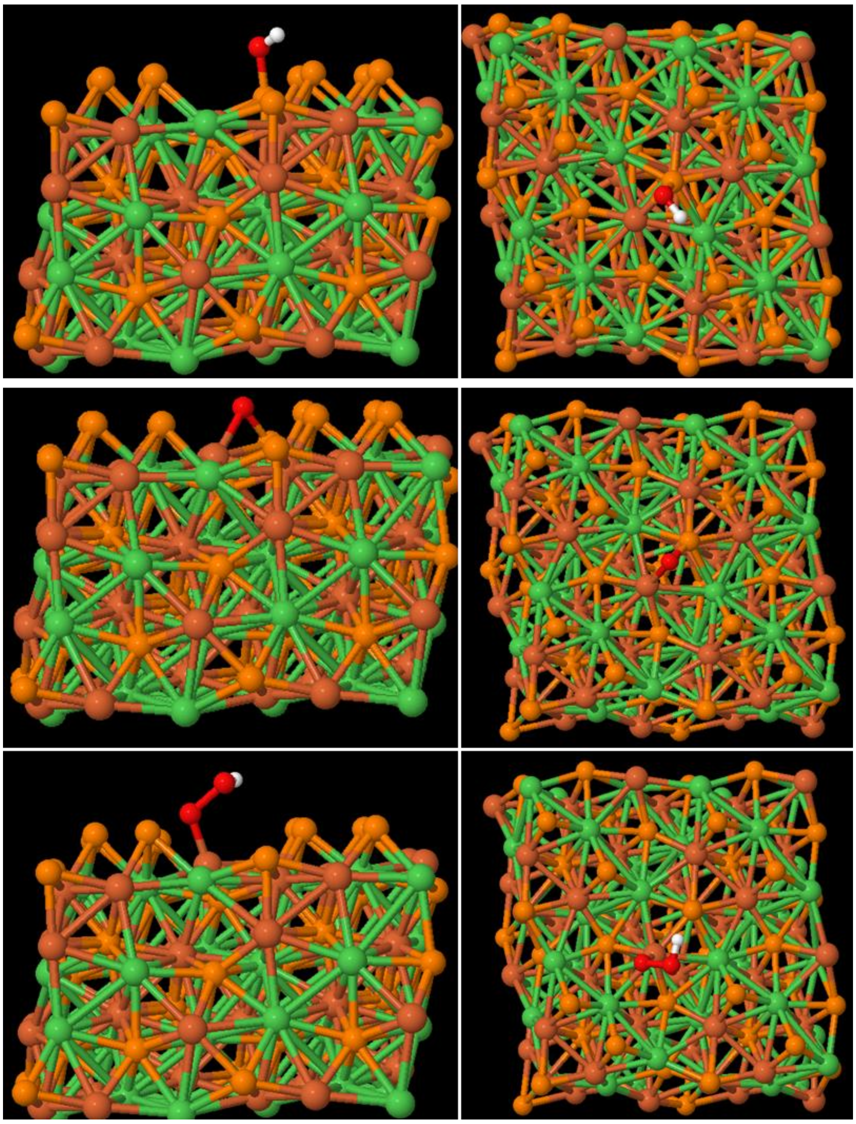

Figure S22. The top and side views of optimized NiFeP (111) of ORR.

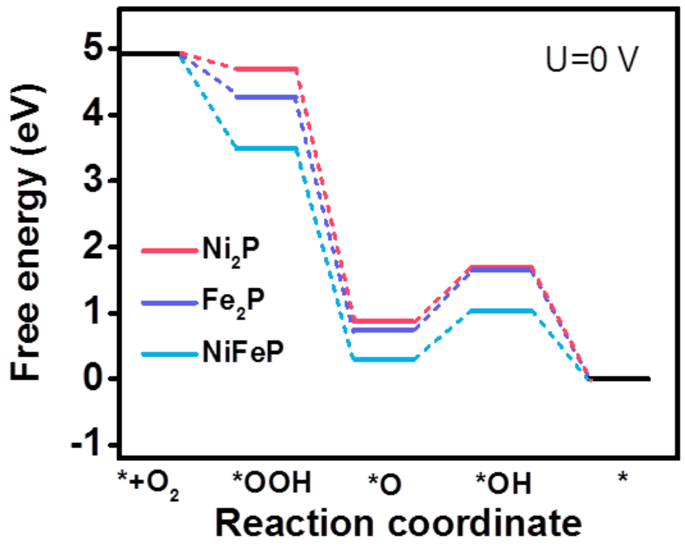

Figure S23. Gibbs free energy (ΔG) plots of the ORR steps on the NiFeP (111), FeP (111), and NiP (111).

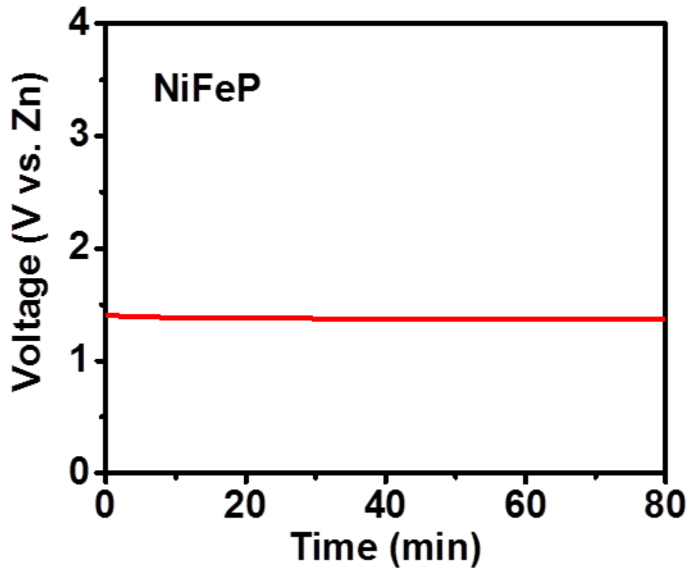

Figure S24. The open-circuit voltage (OCV) of the Zn-air battery with NiFeP as cathode.

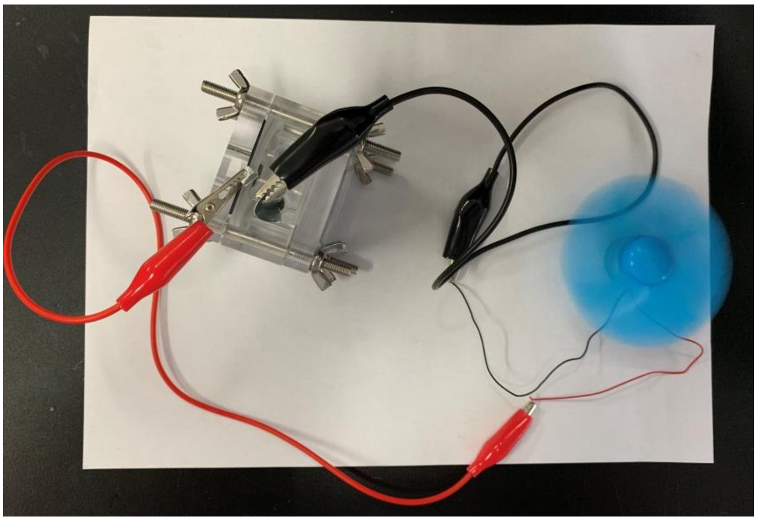

Figure S25. Photograph of pocket fan powered by a Zn-air battery with NiFeP as cathode.

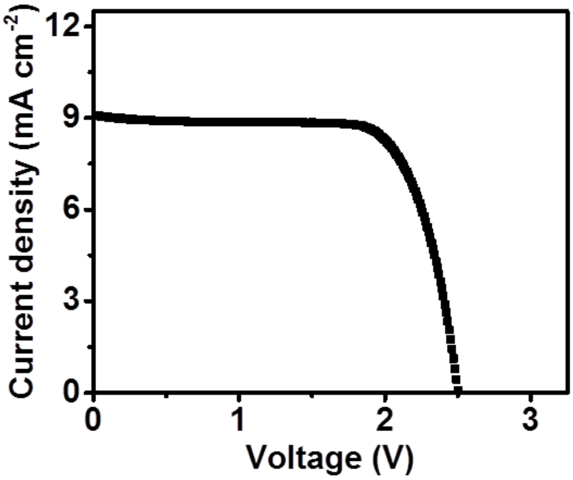

Figure S26. The I-V curve of the solar cell with driving voltage and current under AM 1.5 illumination.

The Si solar cell presented short circuit current density of 9.12 mA cm^{-2} and open circuit potential of 2.5V.

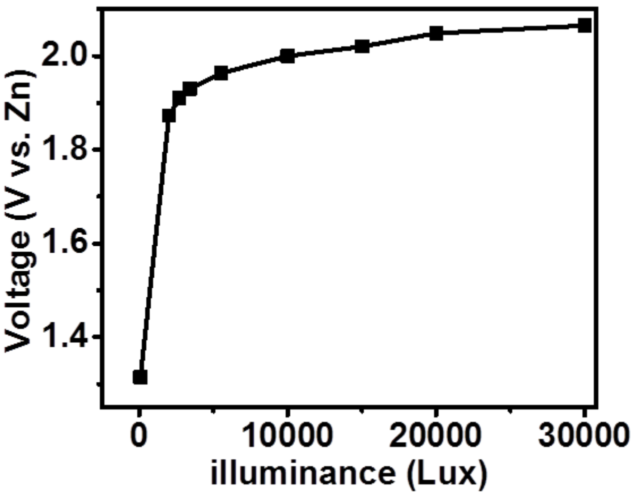

Figure S27. The charge voltage of Zn-air battery with solar cell driven.

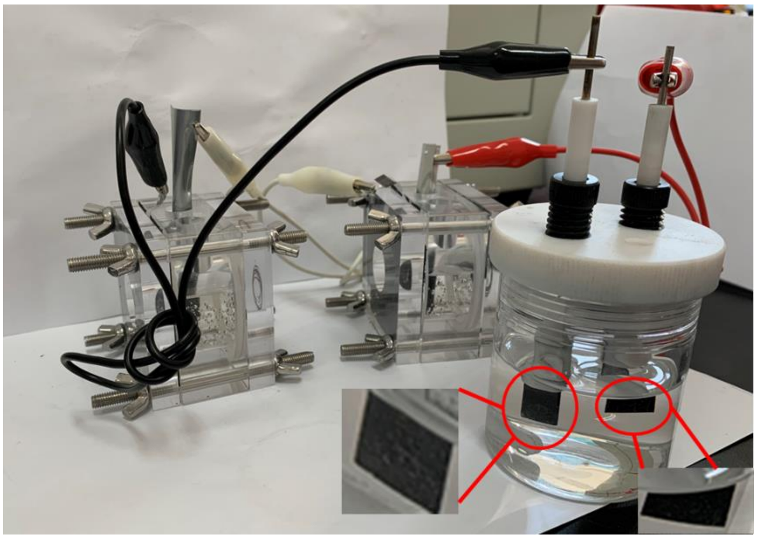

Figure S28. The optical images of the Zn-air battery driven overall water splitting with trifunctional NiFeP. Inset: the bubbles of generated H_2 (left) and O_2 (right).

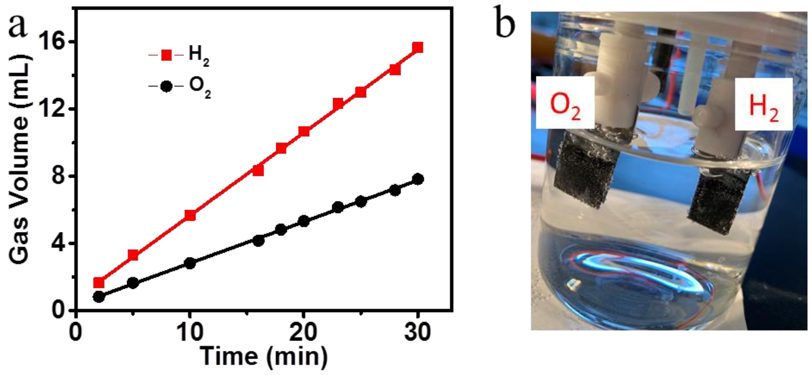

Figure S29. The solar cell directly drove water splitting. (a) The volume of obtained O_2 and H_2. (b) the photo of two electrodes.

Table S1. Comparison of solar-to-hydrogen conversion efficiency.

catalysts	Solar-H_2	references
NiFeP	4.6%	This work
CoPO	0.67%	3
IrCo	1.19%	4
CoP/NGQDsNCs	(AQE) 0.23%	5
FeCoOOH	10.5%	6
NiFe NPs	9.7%	7

For faradic efficiency calculation:

$$\mathrm{FE} = \frac{Q_{H2/O2}}{Q_{total}} = \frac{z * n * \mathrm{F}}{I * t}$$

Notes:

Z: electron transfer number (Z=2 for H_2, 4 for O_2)

n: the amount of substance (mol)

F: Faraday’s constant (96485 C/mol)

I: current (A)

t: time (s)

Zn-battery driven water splitting (data from Figure 4e and 4f):

Current density: 20 mA cm^{-2}

Time:120 min

H_2 volume: 33.2 mL

O_2 volume: 16.4 mL

$$FE_{H2} = \frac{2 * \frac{33.2 * 10^{-3} L}{22.4 \frac{L}{\text{mol}}} * 96485\ C/mol}{0.02 * 2A * 120 * 60\ s} * 100 = 99.3\%$$

$$FE_{O2} = \frac{4 * \frac{16.4 * 10^{-3} L}{22.4 \frac{L}{\text{mol}}} * 96485\ C/mol}{0.02 * 2\ A * 120 * 60\ s} * 100 = 98.1\%$$

Overall water splitting (data from Figure S16):

Current density: 25 mA cm^{-2}

Time:120 min

H_2 volume: 41.7 mL

O_2 volume: 20.5 mL

$$FE_{H2} = 99.8\%$$

$$FE_{O2} = 98.2\%$$

For PV-battery-EC system efficiency:

Light reaction:

$$\eta 1 = \frac{E_{battery\ charged}}{E_{solar}} = \frac{V * I * t}{S * P * t * EE_{solar\ cell}}$$

Notes:

S: area (0.00145 m^2 in our work)

P: 1000 W m^{-2} (the solar irradiation power density on earth at ‘Air Mass 1.5 Global’)[8]

t: reaction time

V: charged voltage of the Zn air battery

I: charged current density

If $EE_{solar\ cell}$=20%

$$\eta 1 = \frac{E_{battery\ charged}}{E_{solar}} = \frac{V * I * t}{S * P * t * EE_{solar\ cell}}$$

$$= \frac{1.898\ V * 11.11 \frac{mA}{cm^2} * 10^{-3} * 1\ cm^2 * 120 * 60\ s}{0.00145\ m^2 * 1000 \frac{\text{W}}{\text{m2}} * 120 * 60\ \text{s} * 20\%} * 100 = 7.27\%$$

Unassisted light reaction:

Zn air-to-water splitting efficiency:

$$\eta 2 = \frac{E_{water\ splitting}}{E_{battery\ discharge}} = \frac{\text{U} * \text{I} * \text{t}}{V * I * t} = \frac{1.9\ V * 40\text{mA} * 120 * 60\text{s}}{1.16V * 2 * 40\text{mA} * 120 * 60\text{s}} * 100 = 82\%$$

Note:

U: voltage of water splitting

Zn air-to-hydrogen efficiency:

$$\eta 3=\frac{E(H_2)}{E_{battery\ discharge}}=\frac{\text{standard molar enthalpy of combustion }\left(\frac{\text{kJ}}{\text{mol}}\right)*\ H_2(\text{mol})}{V*I*t}$$

$$=\frac{285*\frac{10^3\text{J}}{\text{mol}}*\ 33.2\text{ mL}*\frac{10^{-3}}{22.4}L/mol}{1.16V*2*40mA*10^{-3}*120*60}*100=63.2\%$$

Note: The energy of H_2 was obtained according to the standard molar enthalpy of combustion (-285 kJ/mol)[9].

Solar-to –hydrogen efficiency:

$$\eta=\frac{E_{battery\ charged}}{E_{solar}}*\frac{E_{water\ splitting\ driven\ by\ battery}(H_2)}{E_{battery\ discharge}}=\eta 1*\eta 3=7.27\%*63.2\%$$

$$=4.6\%$$

Self-powered system efficiency (solar-to-water splitting device):

$$\eta=\ \eta 1*\eta 2=7.27\%*82\%=5.9\%$$

For PV-EC system efficiency:

According to the reported works[10, 11, 12], there have two ways to calculate the solar-to-hydrogen efficiency.
One way: according to the standard molar enthalpy of combustion (-285 kJ mol^{-1})

$$\eta=\frac{\text{standard molar enthalpy of combustion (KJ mol}^{-1}\text{)}\times \text{H}_2\text{(mol)}}{\text{illumination power (W)}\times \text{time(s)}}$$

$$=\frac{-285\text{KJ mol}^{-1}\times 15.67\text{ mL}\times\frac{10^{-3}}{22.4}\text{L/mol}}{0.00145\text{ m}^2\times 1000\text{W/m}^2\times 30\times 60\text{s}}=7.6\%$$

The other way: according to the thermodynamic potential (1.23 V) of water splitting, FE_{H2}=99%

$$\eta=\frac{\text{J (A)}\times 1.23\text{ V}\times \text{FE}}{\text{illumination power (W)}}$$

$$=\frac{0.075\text{ mA}\times 1.23\text{ V}\times 99\%}{\text{illumination power (W)}}=6.3\%$$

References:

[1]. Yanping Zhu, Gao Chen, Xiaomin Xu, Guangming Yang, Meilin Liu, Zongping Shao, ACS Catal. **2017**, 7, 3540.

[2]. Huang, Y. Y.; Yang, R.; Anandhababu, G.; Xie, J. F.; Lv, J. Q.; Zhao, X. T.; Wang, X. Y.; Wu, M. X.; Li, Q. H.; Wang, Y. B., ACS Energy Lett. **2018**, 3, 1854-1860.

[3]. Anandhababu, G.; Huang, Y.; Babu, D. D.; Wu, M.; Wang, Y., Adv. Funct. Mater. **2018**, 1706120.

[4]. Babu, D. D.; Huang, Y.; Anandhababu, G.; Wang, X.; Si, R.; Wu, M.; Li, Q.; Wang, Y.; Yao, J., J.

Mater. Chem. A **2019,** *7*, 8376-8383.

[5]. Zhong, Z.; Liu, J.; Xu, X.; Cao, A.; Tao, Z.; You, W.; Kang, L., J. Mater. Chem. A **2021,** *9*, 2404-2413.

[6]. Lv, J.; Guan, X.; Huang, Y.; Cai, L.; Yu, M.; Li, X.; Yu, Y.; Chen, D., Nanoscale **2021,** *13*, 15755-15762.

[7]. Kumar, A.; Chaudhary, D. K.; Parvin, S.; Bhattacharyya, S. J. Mater. Chem. A **2018**, 6, 18948-18959.

[8]. Qi, J.; Zhang, W.; Cao, R., Adv. Energy Mater. **2018**, 8, 1701620.

[9]. Li, J.; Chu, D.; Baker, D. R.; Leff, A.; Zheng, P.; Jiang, R, ACS Applied Energy Materials **2021**, 4, 9969-9981.

[10]. Chen, H.; Song, L.; Ouyang, S.; Wang, J.; Lv, J.; Ye, J., Adv Sci (Weinh) **2019**, 6, 1900465.

[11]. Lv, J.; Guan, X.; Huang, Y.; Cai, L.; Yu, M.; Li, X.; Yu, Y.; Chen, D., Nanoscale **2021**, 13, 15755-15762.

[12]. Nordmann, S.; Berghoff, B.; Hessel, A.; Zielinsk, B.; John, J.; Starschich, S.; Knoch, J., Sol. Energy Mater. Sol. Cells **2019**, 191, 422-426.
