# Supplementary material for: A Trifunctional Ni–P/Fe–P Collaborated Electrocatalyst Enables Self‐Powered Energy Systems
Source: Adv Sci (Weinh). 2022 May 22;9(22):2201594. doi: 10.1002/advs.202201594 (PMC9353458; doi:10.1002/advs.202201594)
Supplement: Supplementary file 2 — Supporting Information [file ADVS-9-2201594-s001.pptx]

## Slide 1
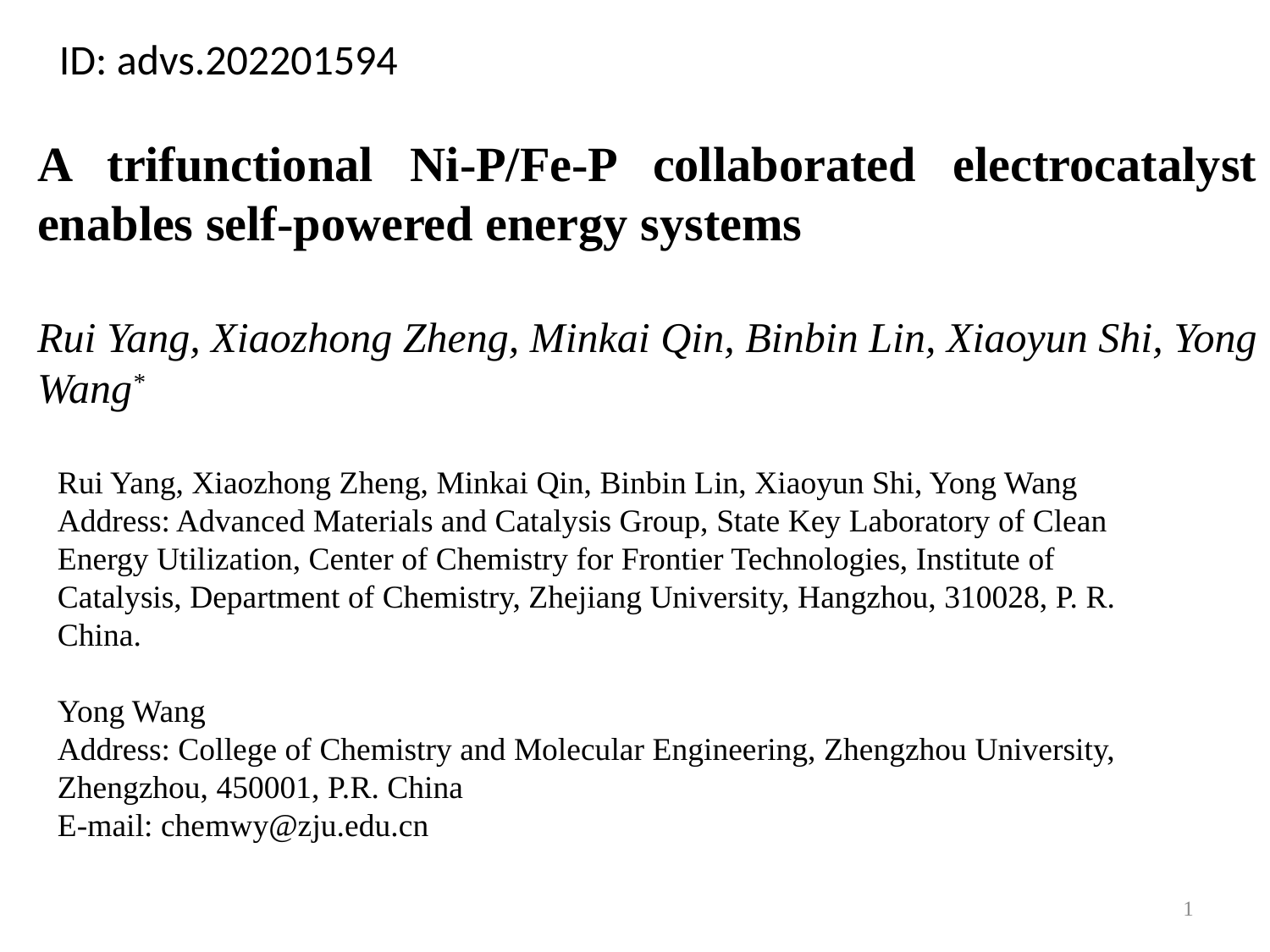

ID: advs.202201594
A trifunctional Ni-P/Fe-P collaborated electrocatalyst enables self-powered energy systems
Rui Yang, Xiaozhong Zheng, Minkai Qin, Binbin Lin, Xiaoyun Shi, Yong Wang*
Rui Yang, Xiaozhong Zheng, Minkai Qin, Binbin Lin, Xiaoyun Shi, Yong Wang
Address: Advanced Materials and Catalysis Group, State Key Laboratory of Clean Energy Utilization, Center of Chemistry for Frontier Technologies, Institute of Catalysis, Department of Chemistry, Zhejiang University, Hangzhou, 310028, P. R. China.
Yong Wang
Address: College of Chemistry and Molecular Engineering, Zhengzhou University, Zhengzhou, 450001, P.R. China
E-mail: chemwy@zju.edu.cn
1

## Slide 2
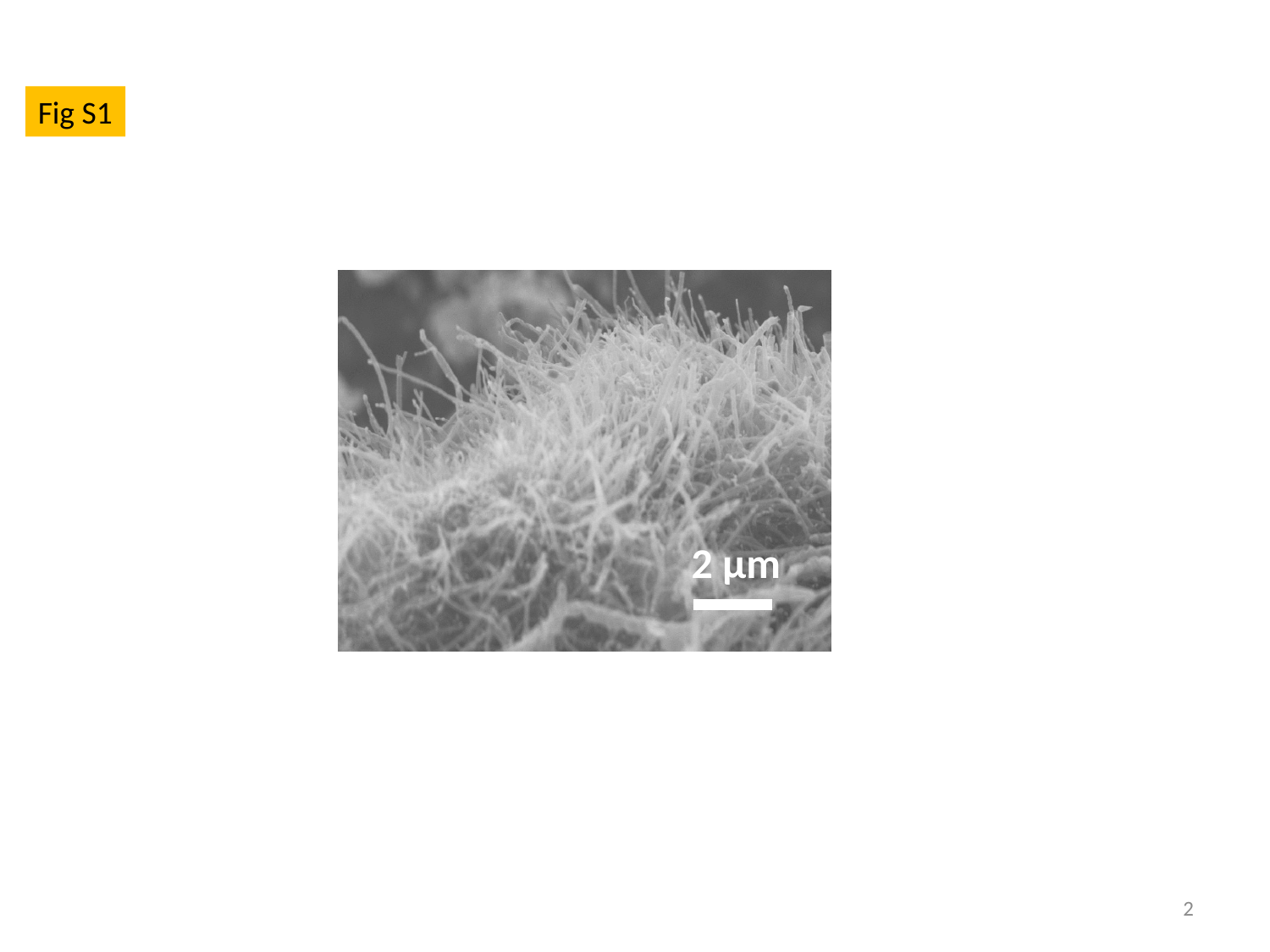

Fig S1
2 μm
2

## Slide 3
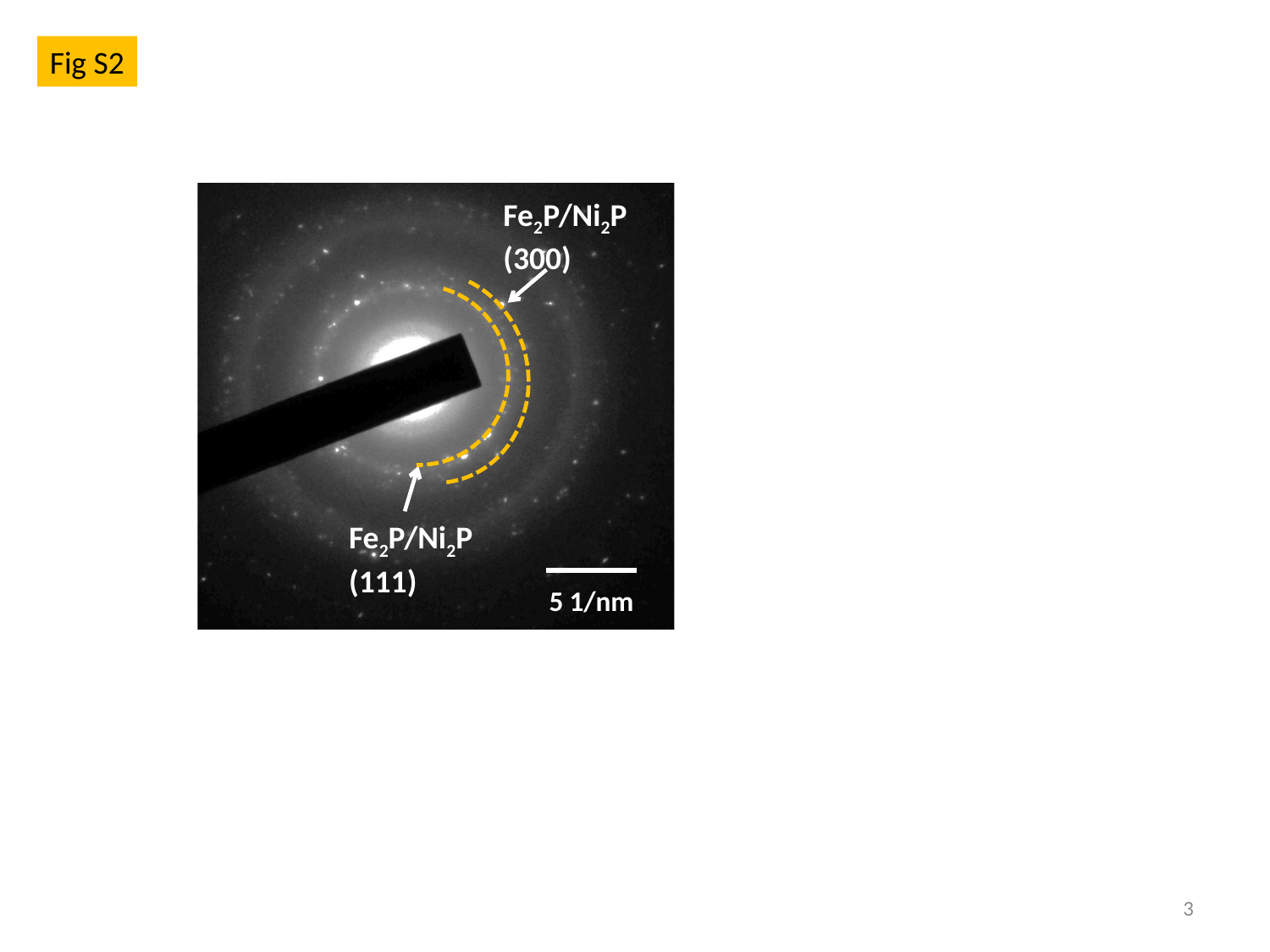

Fig S2
Fe2P/Ni2P
(111)
Fe2P/Ni2P
(300)
5 1/nm
3

## Slide 4
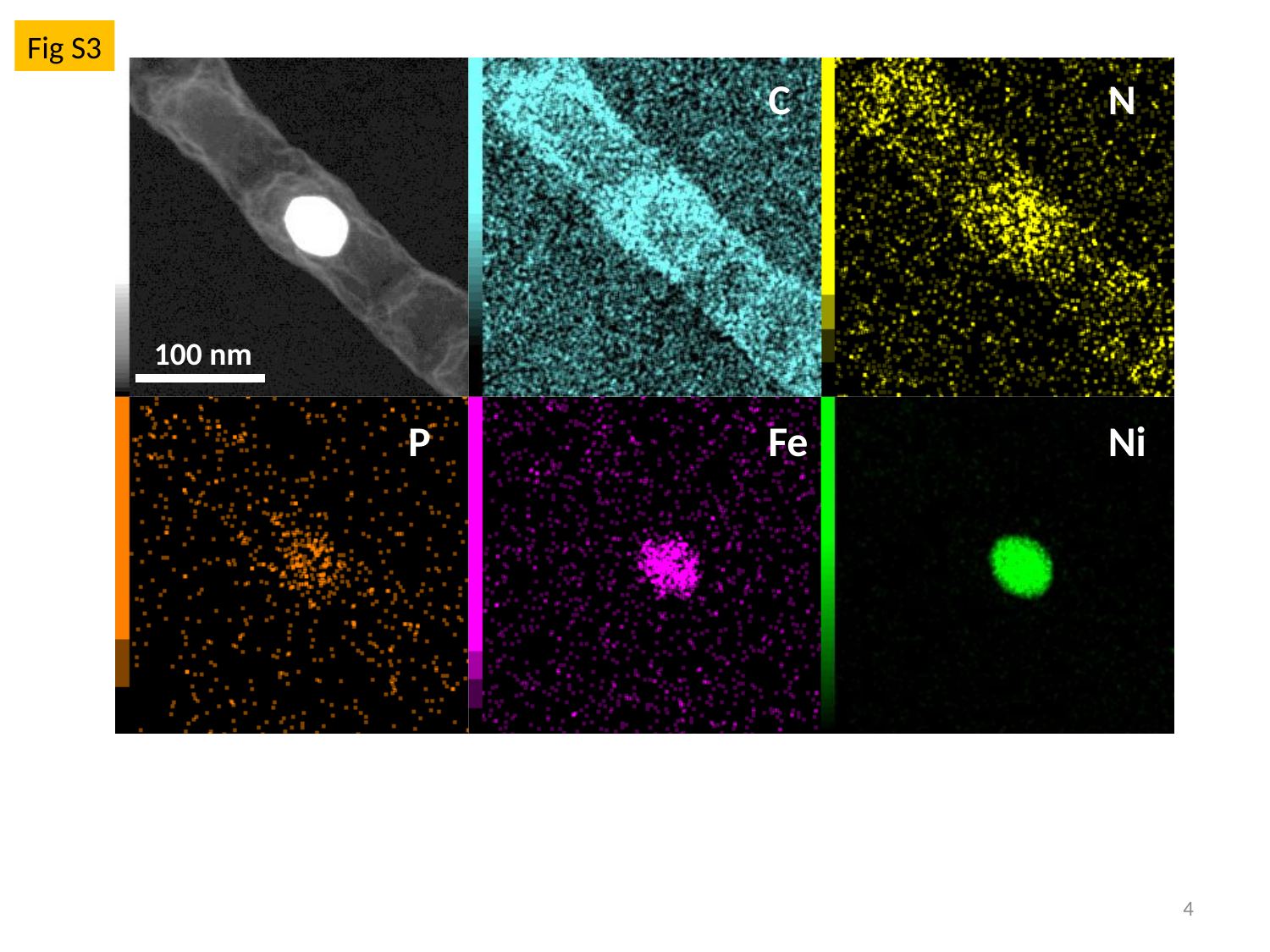

Fig S3
C
N
100 nm
P
Fe
Ni
4

## Slide 5
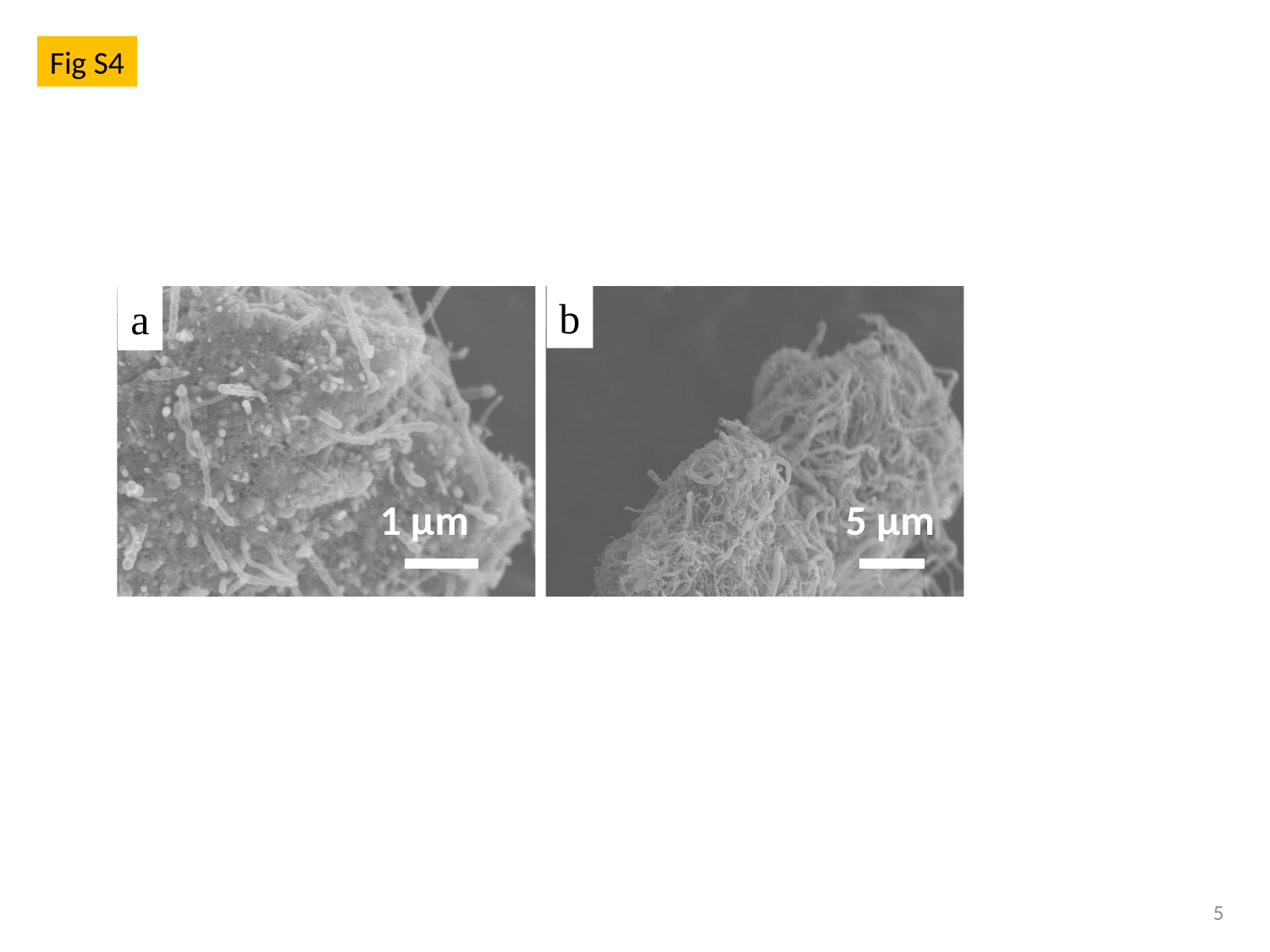

Fig S4
b
a
1 μm
5 μm
5

## Slide 6
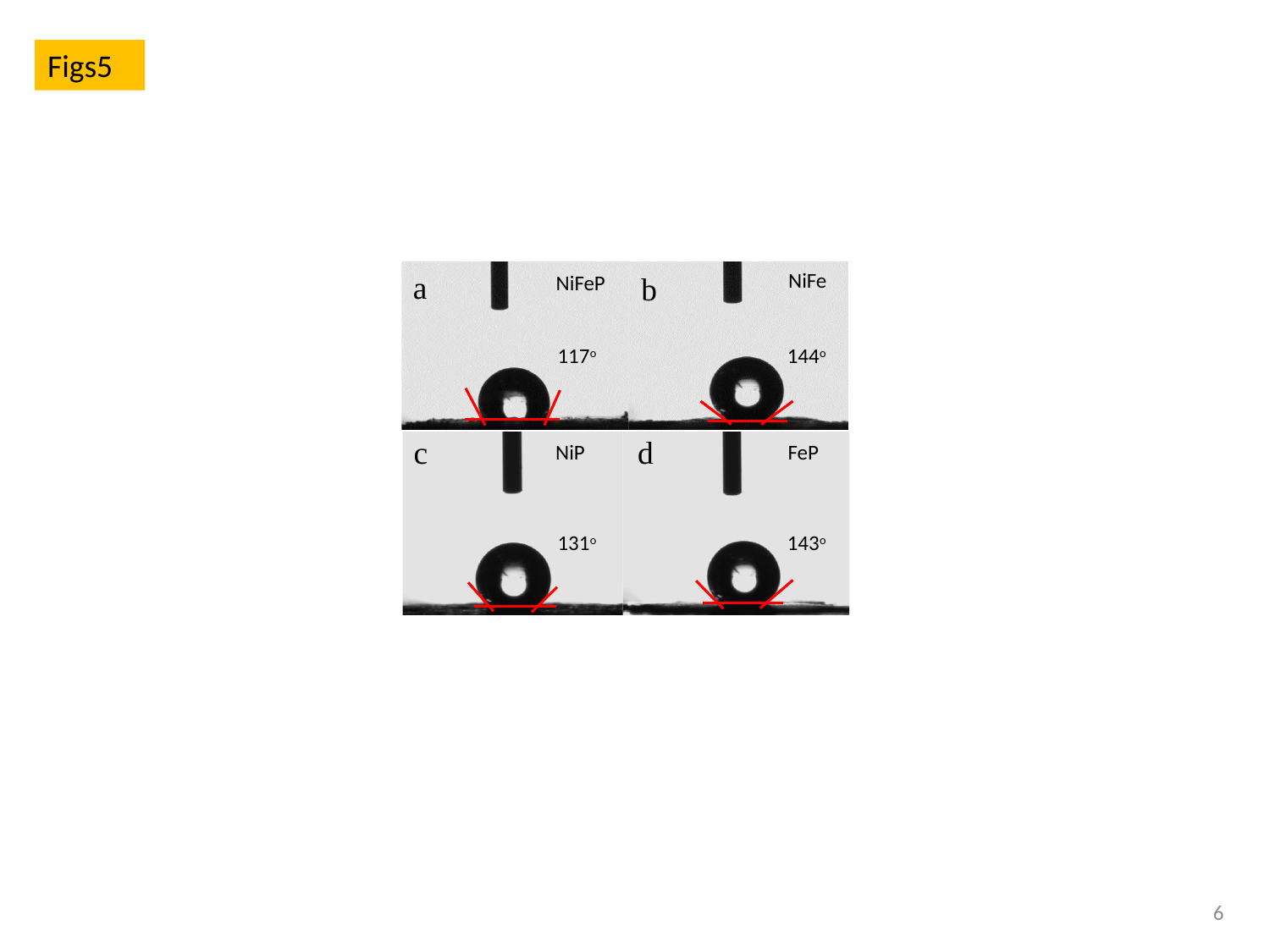

Figs5
NiFe
a
NiFeP
b
c
d
NiP
FeP
117o
144o
131o
143o
6

## Slide 7
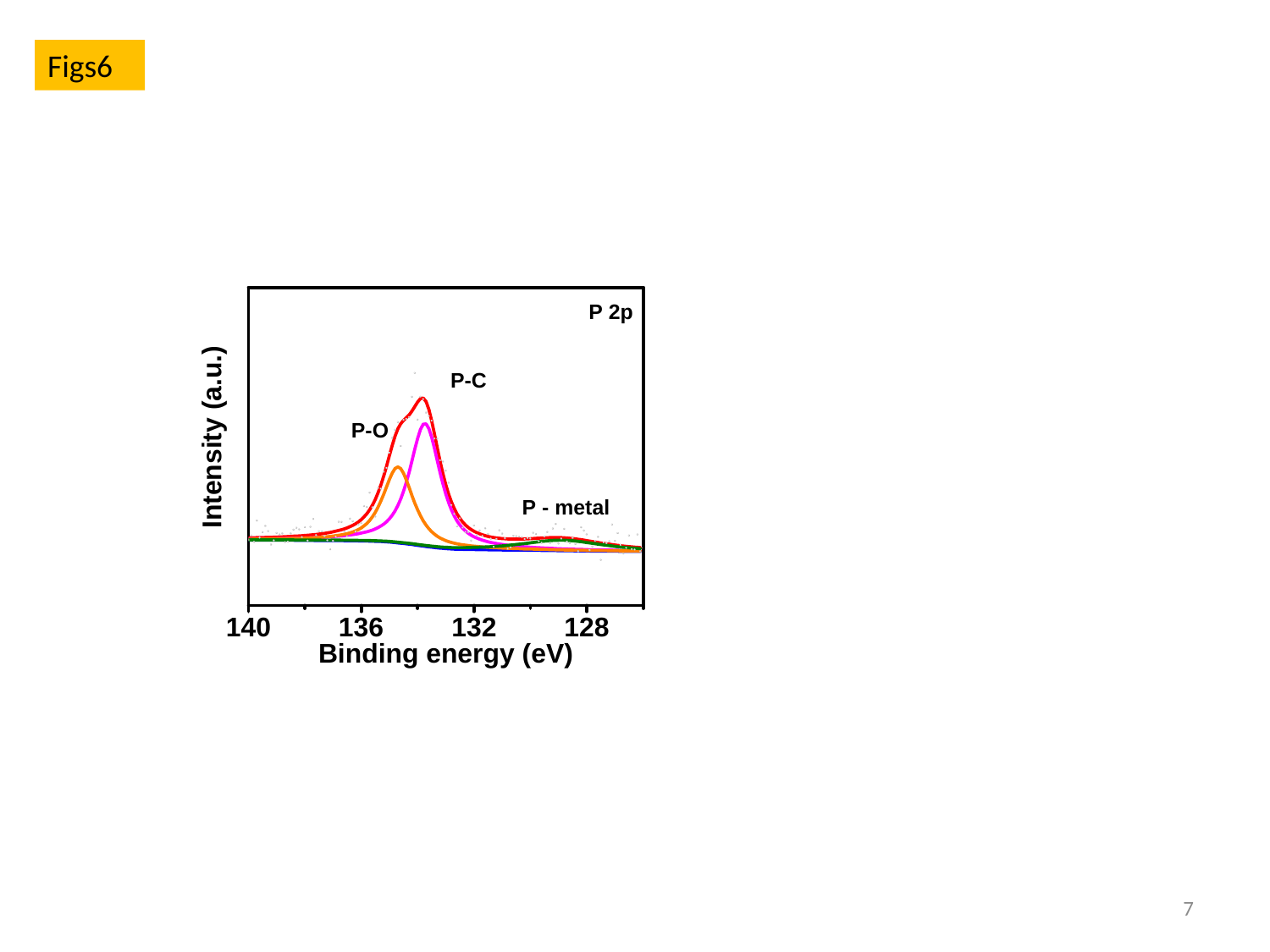

Figs6
7

## Slide 8
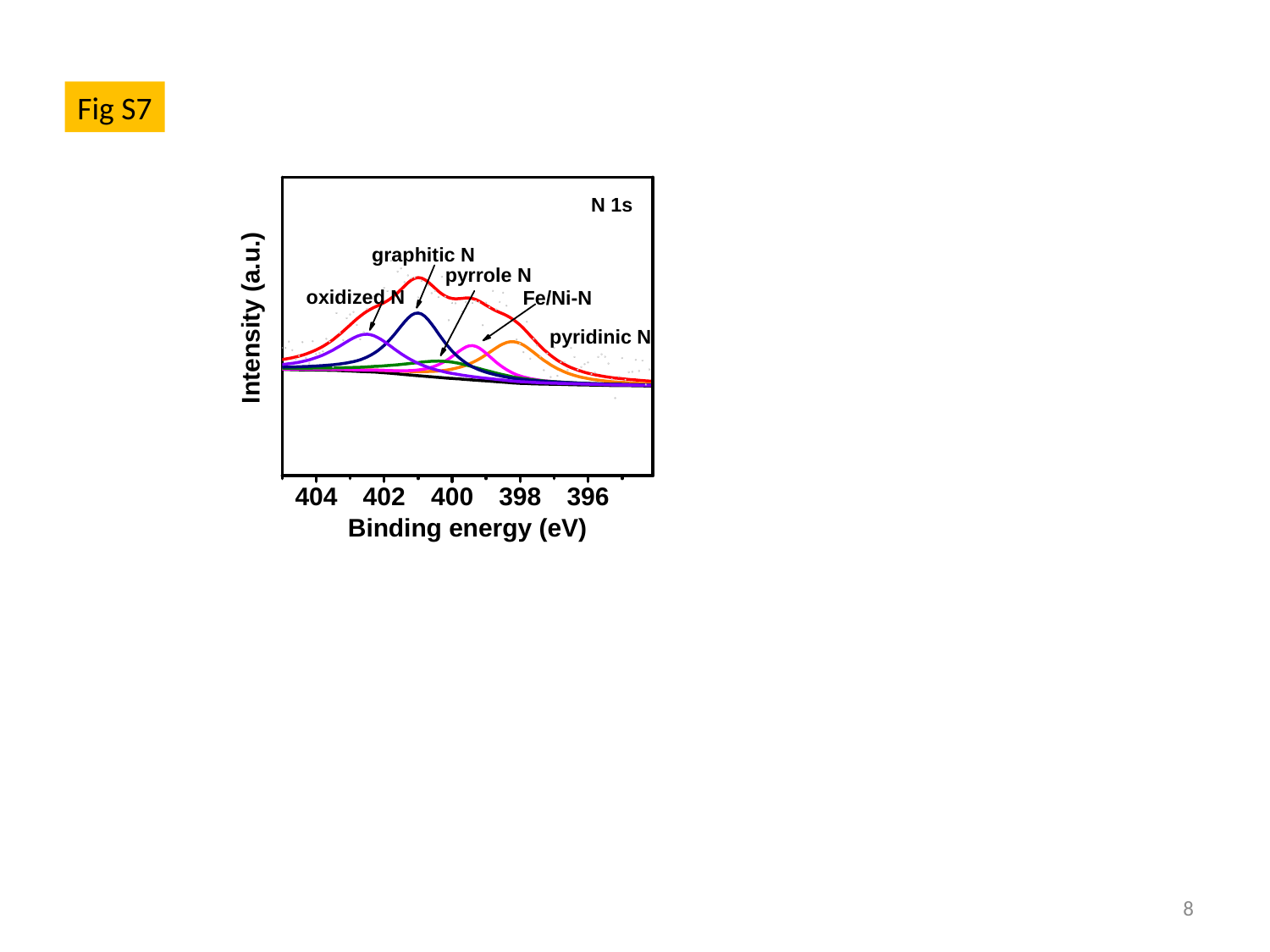

Fig S7
8

## Slide 9
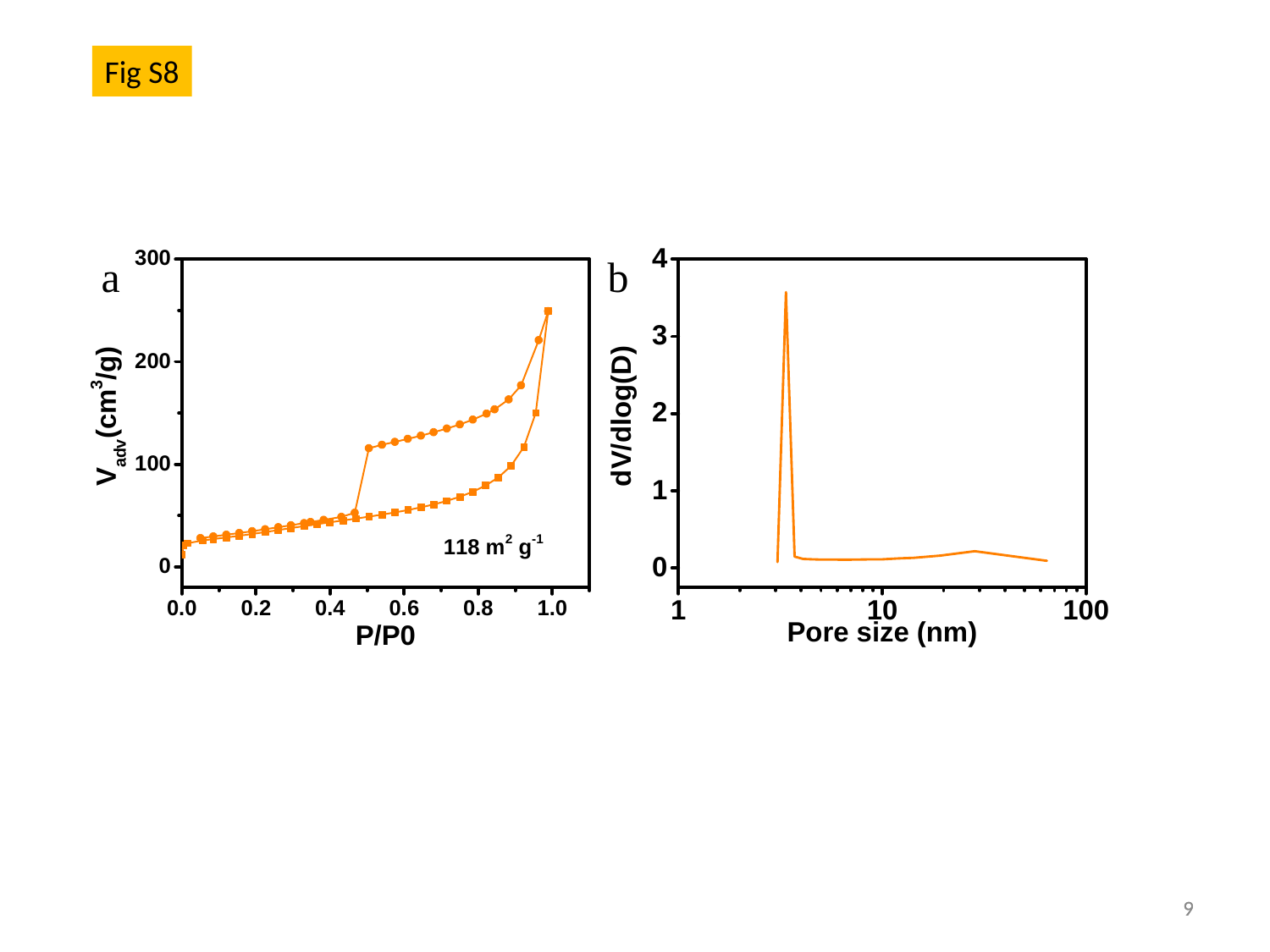

Fig S8
a
b
9
9

## Slide 10
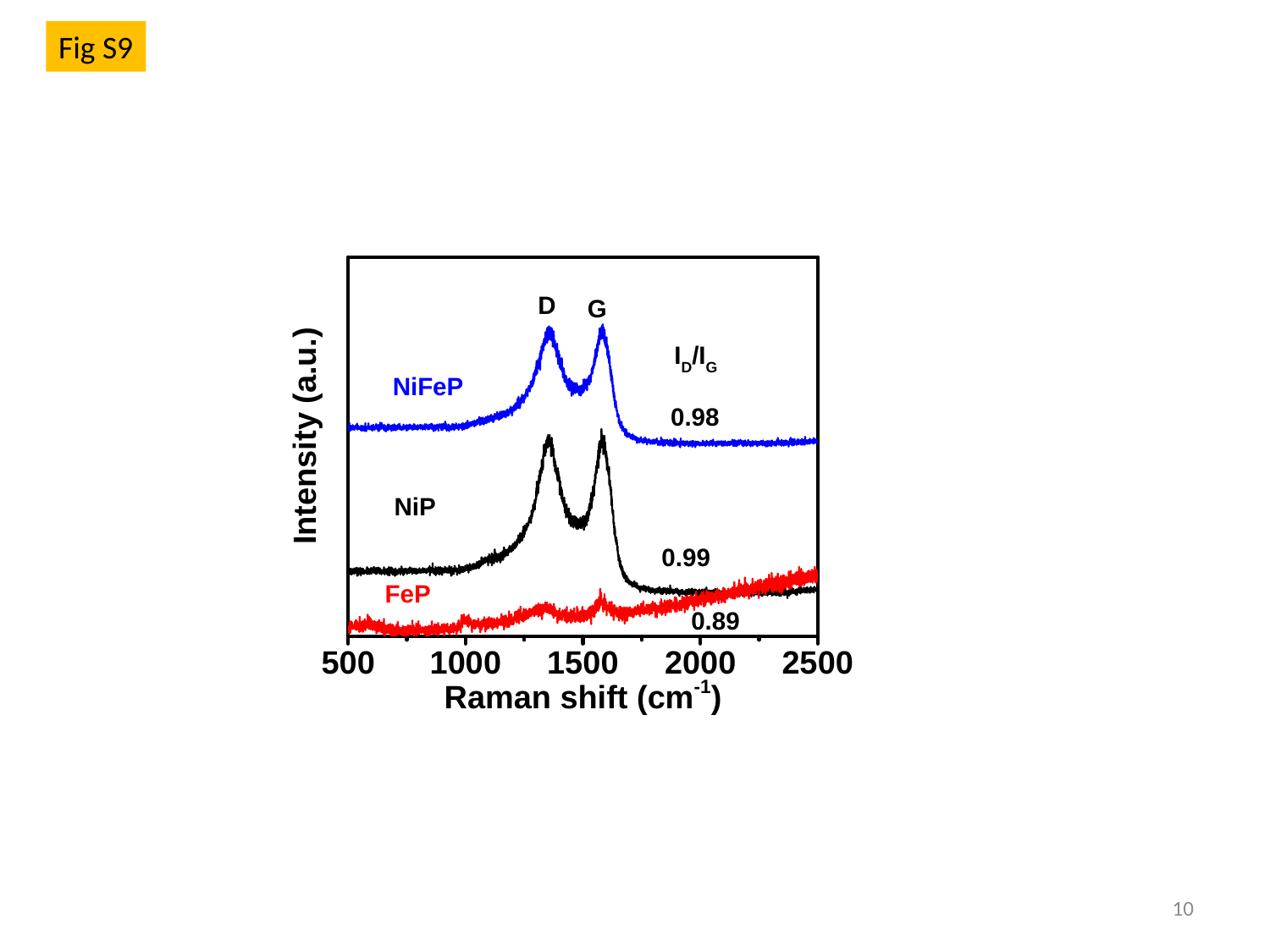

Fig S9
10

## Slide 11
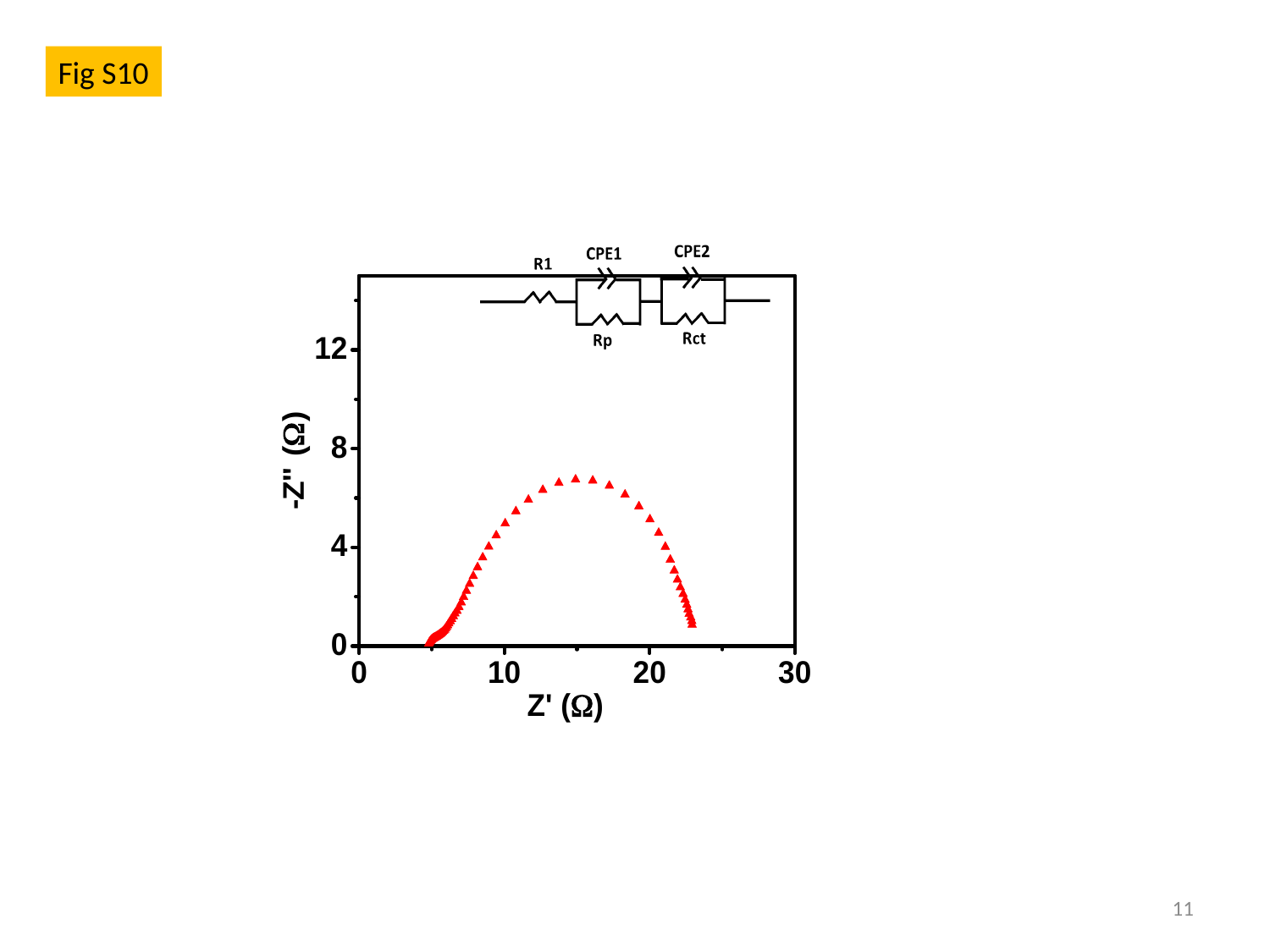

Fig S10
11

## Slide 12
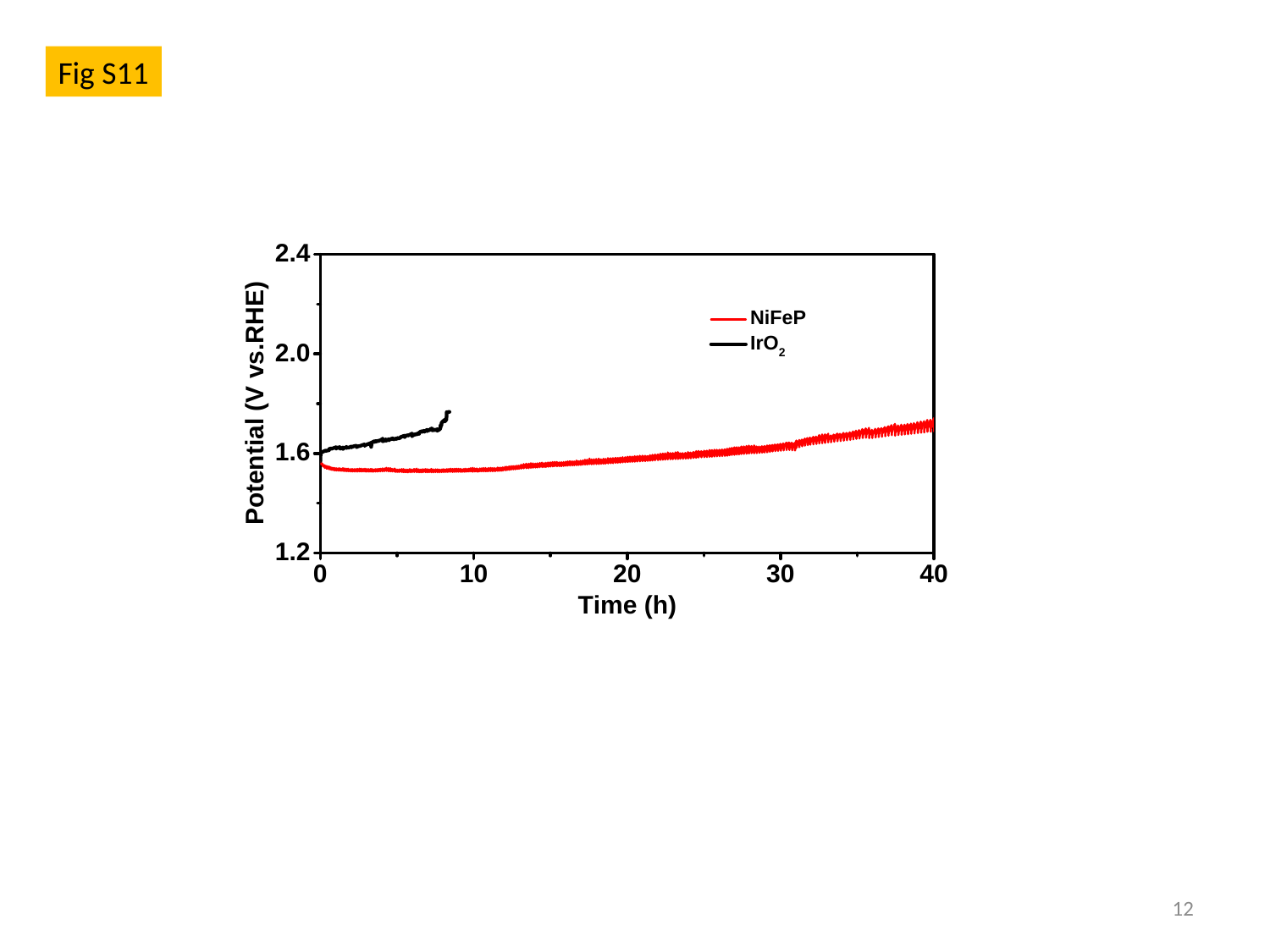

Fig S11
12

## Slide 13
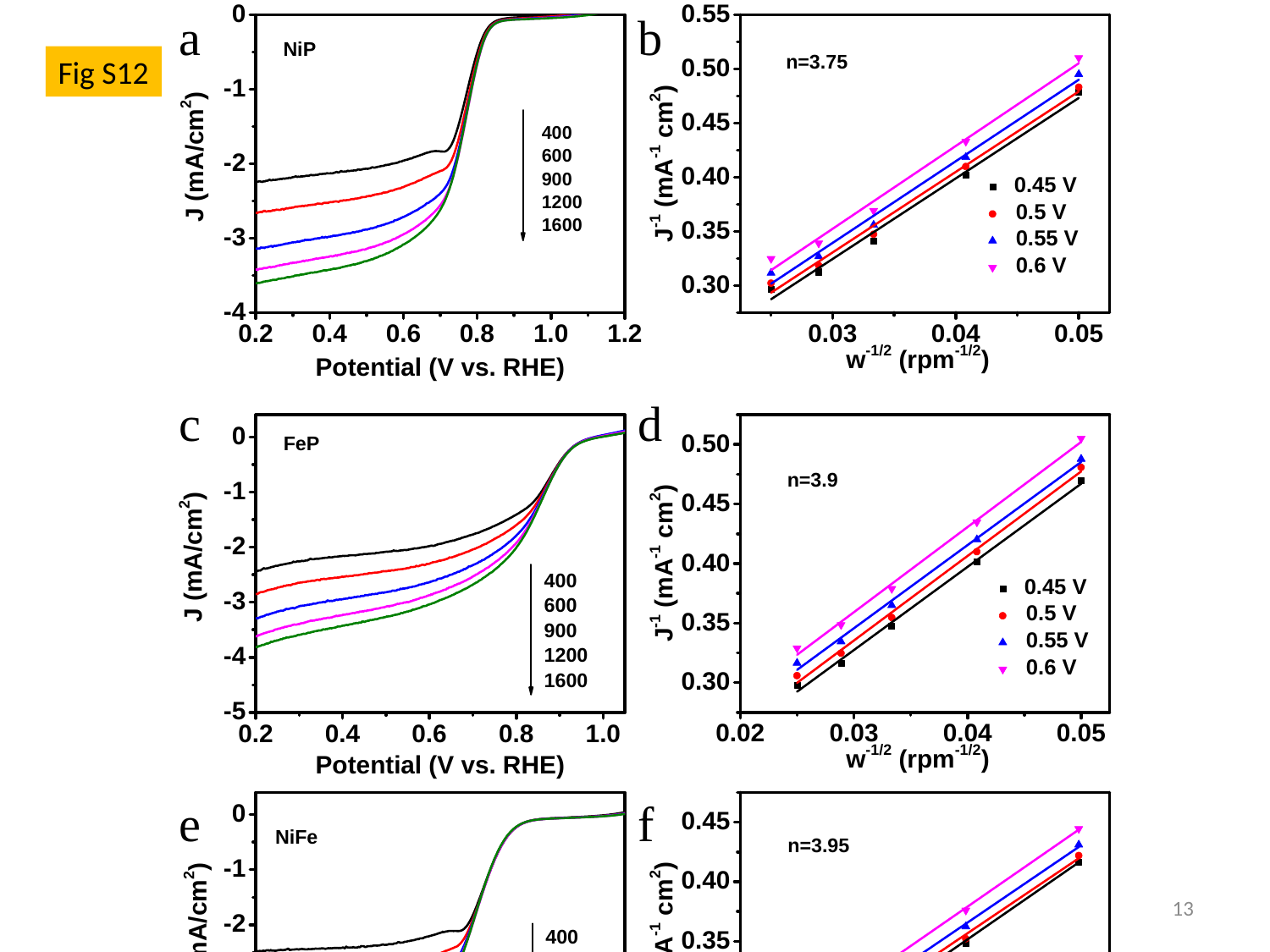

a
b
c
d
e
f
Fig S12
13

## Slide 14
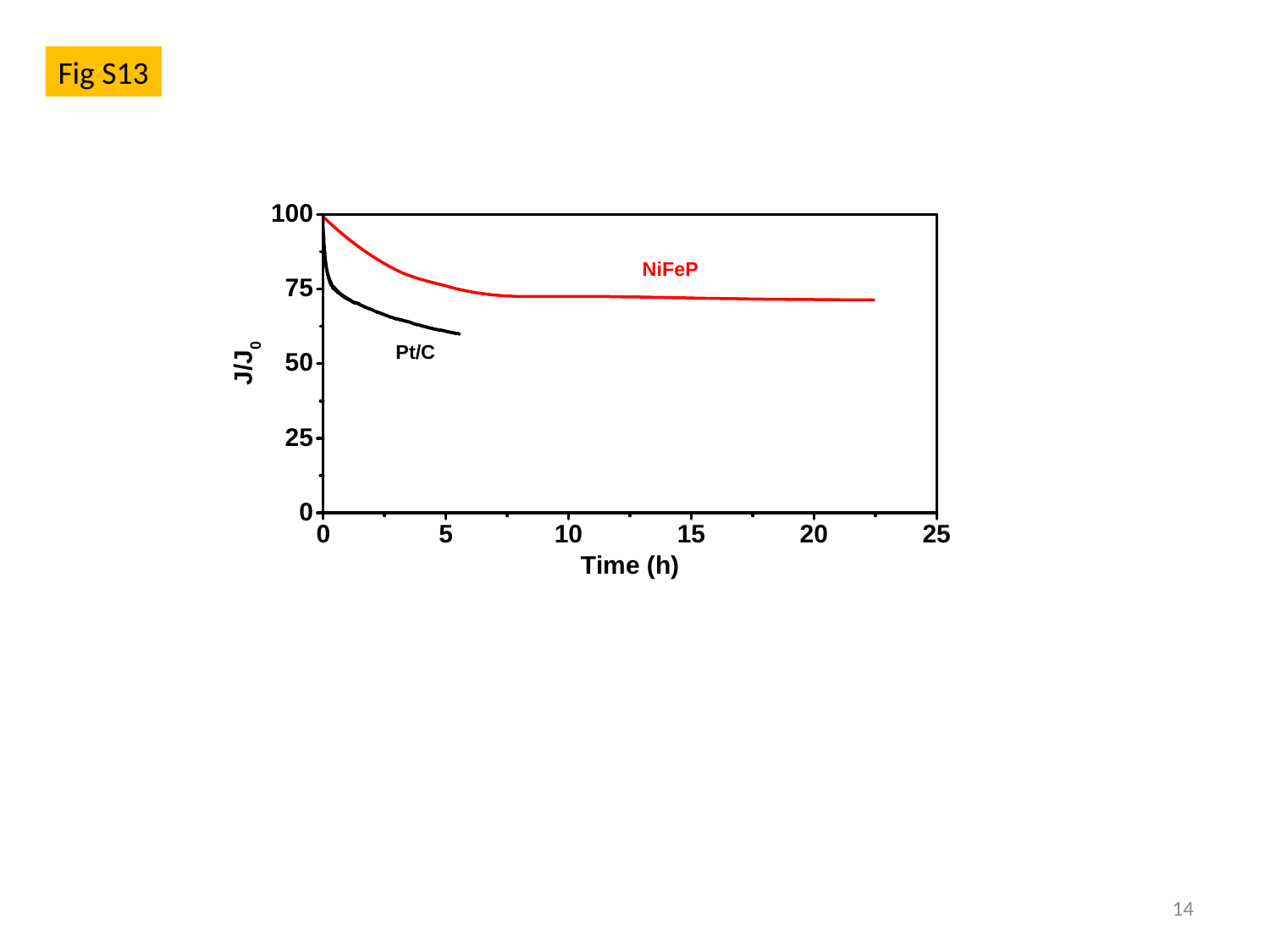

Fig S13
14

## Slide 15
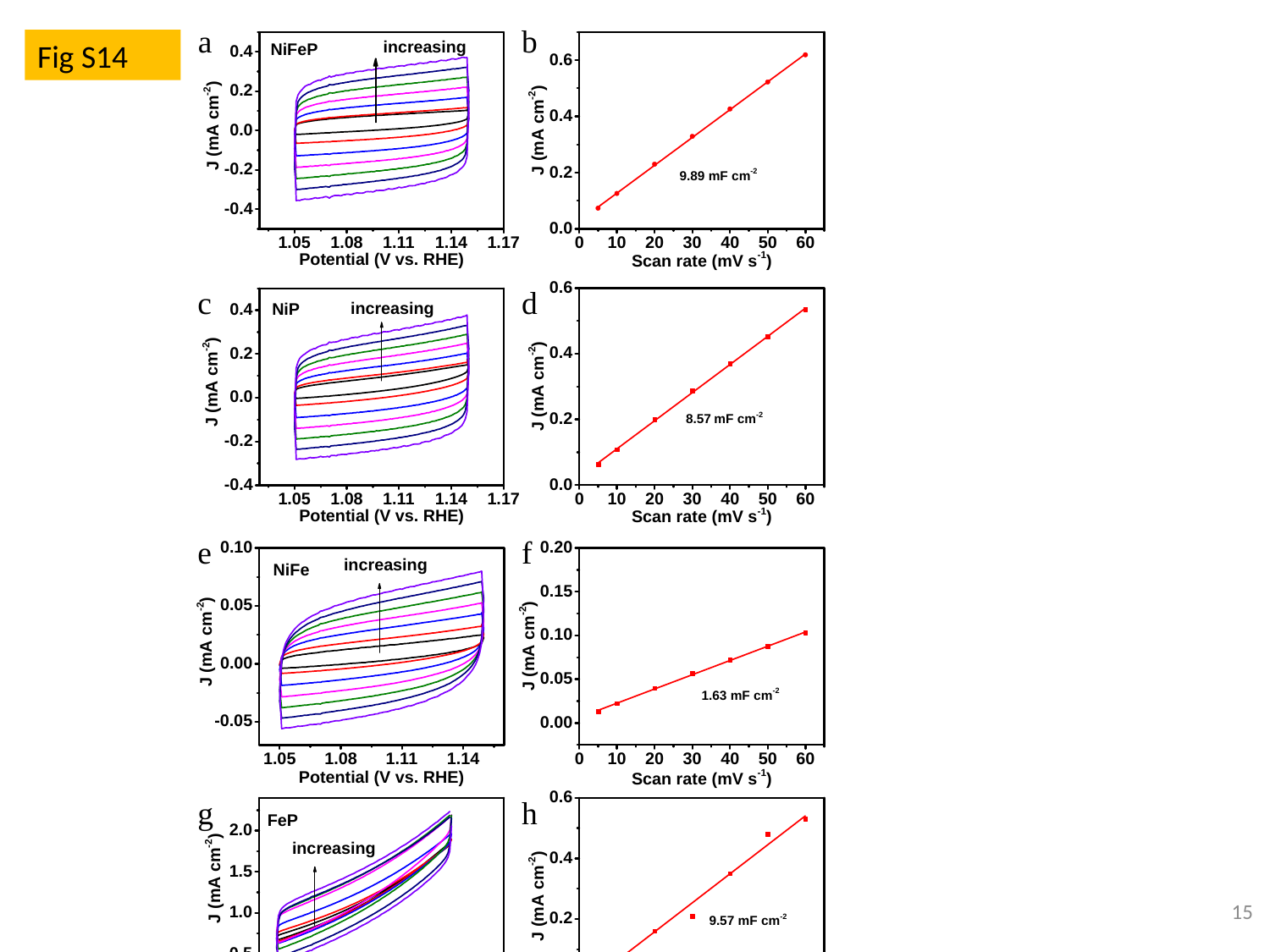

a
b
c
d
e
f
g
h
Fig S14
15

## Slide 16
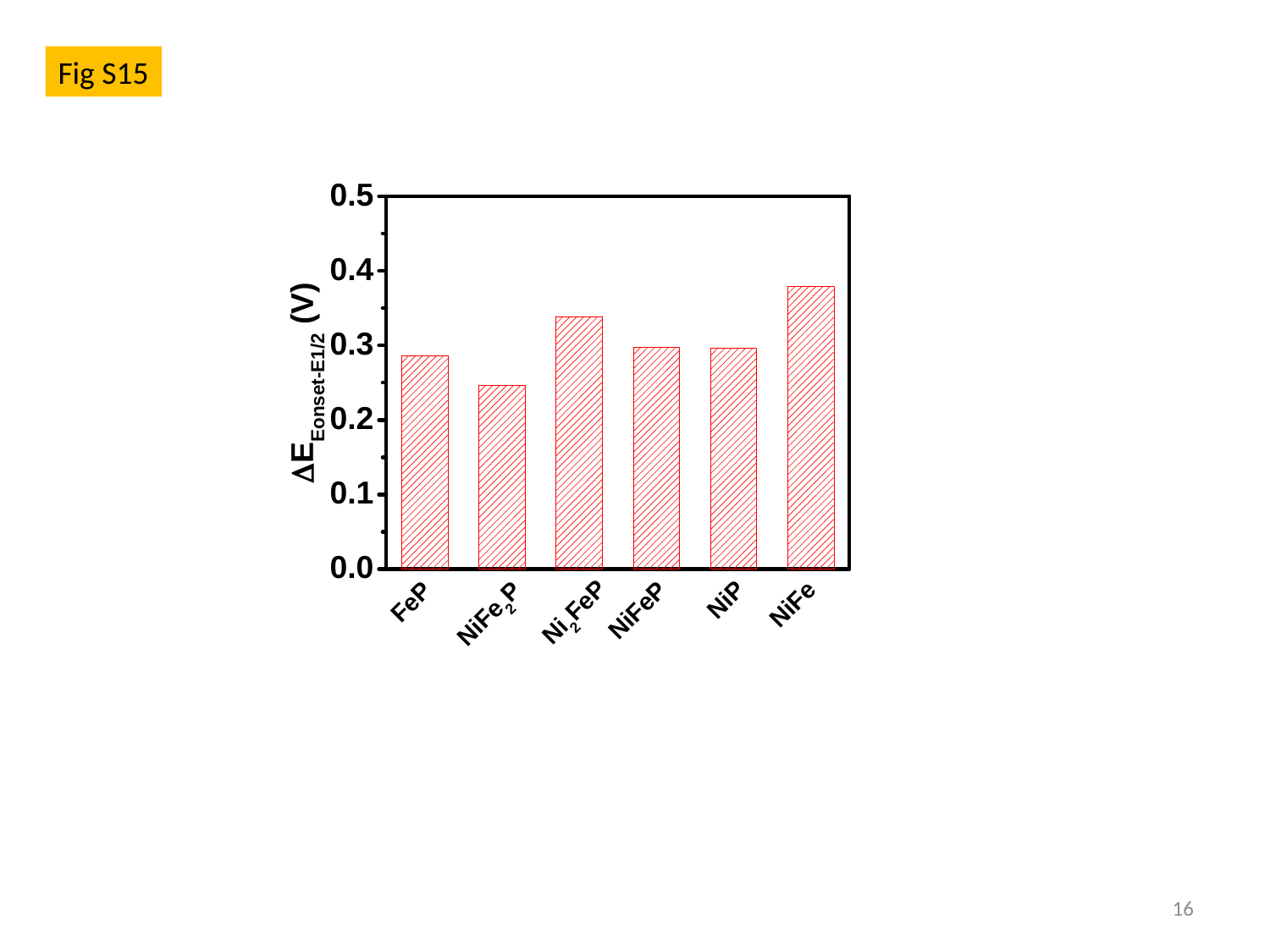

Fig S15
16

## Slide 17
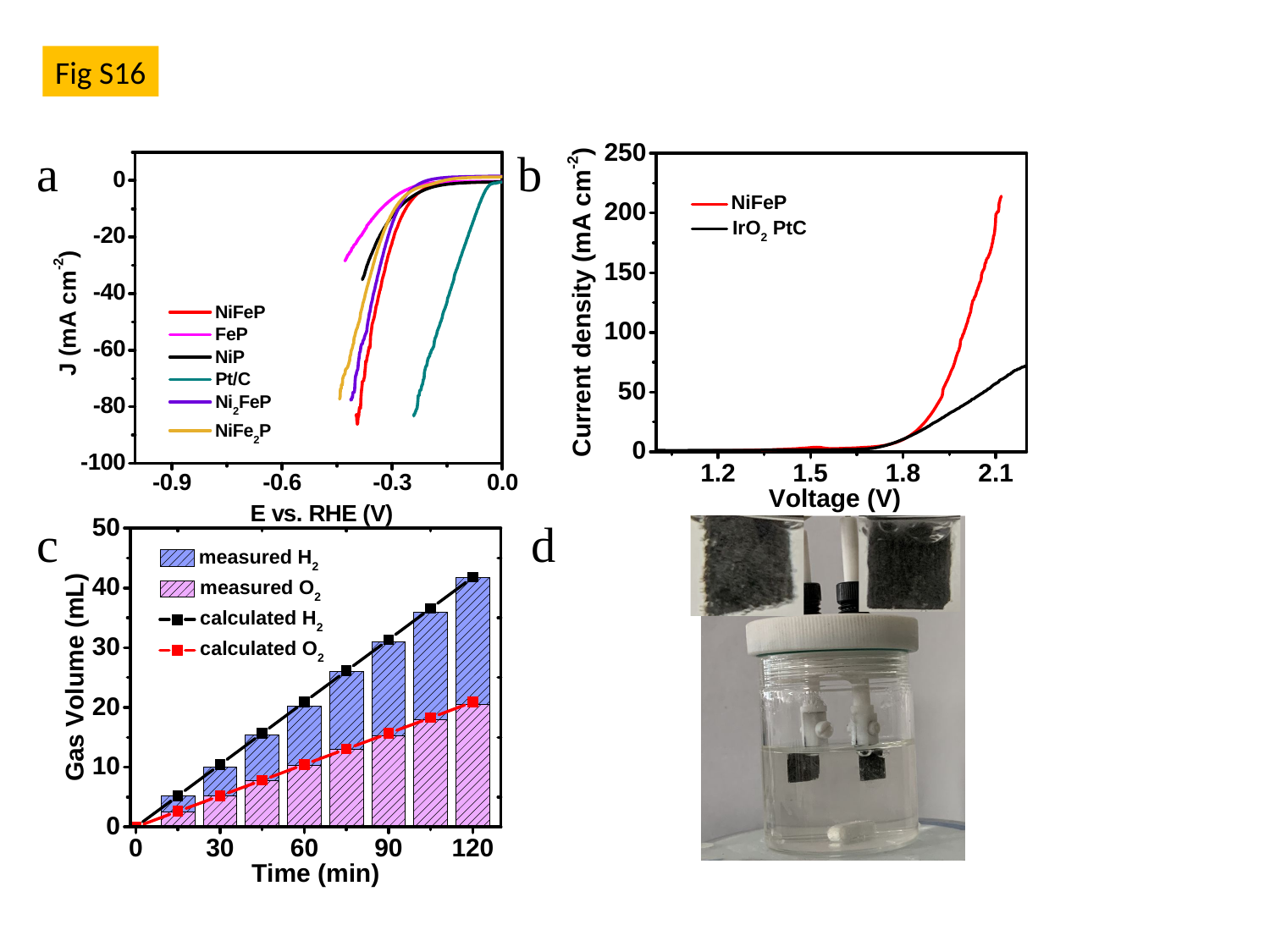

Fig S16
a
b
c
d
O2
H2

## Slide 18
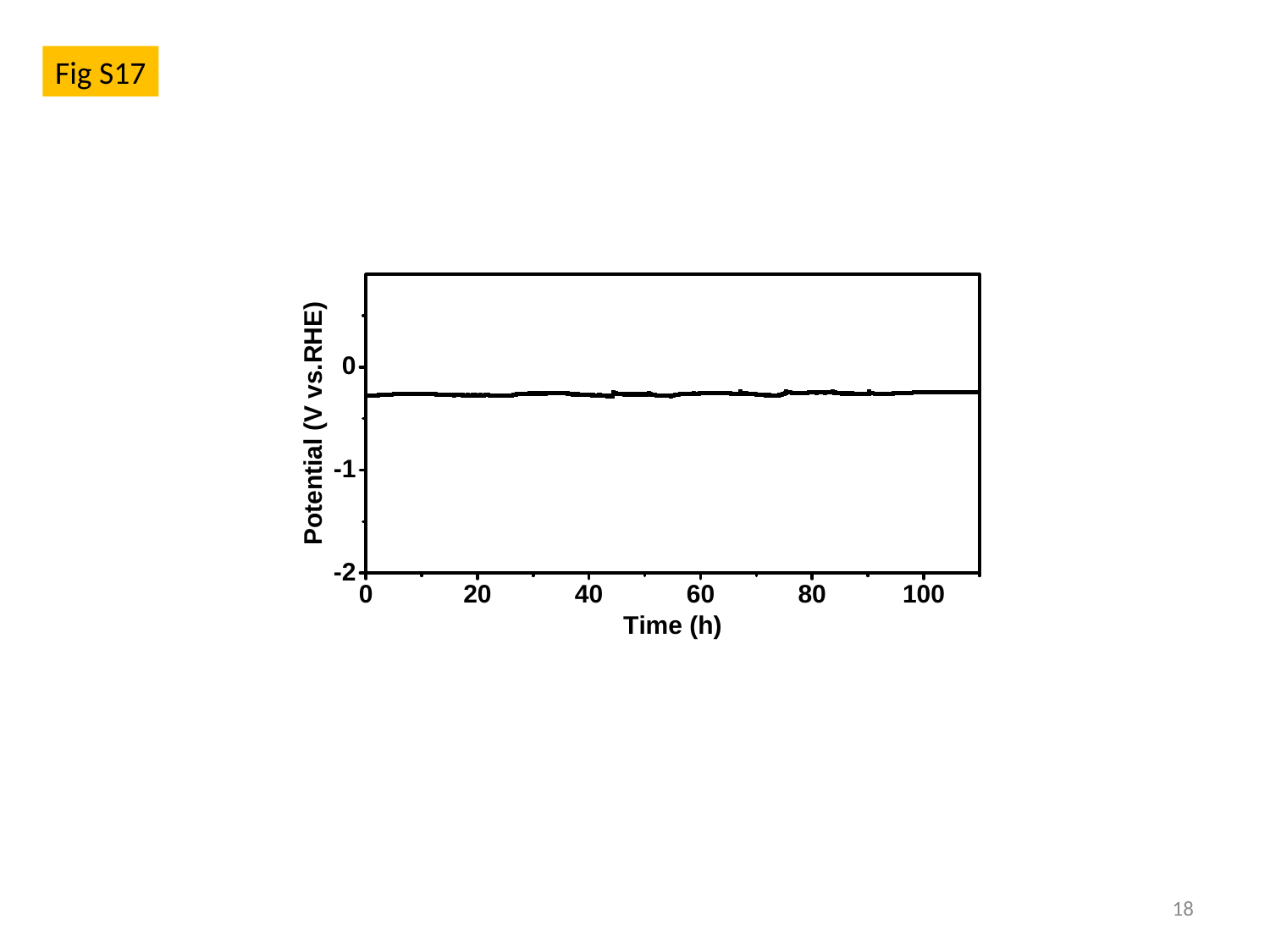

Fig S17
18

## Slide 19
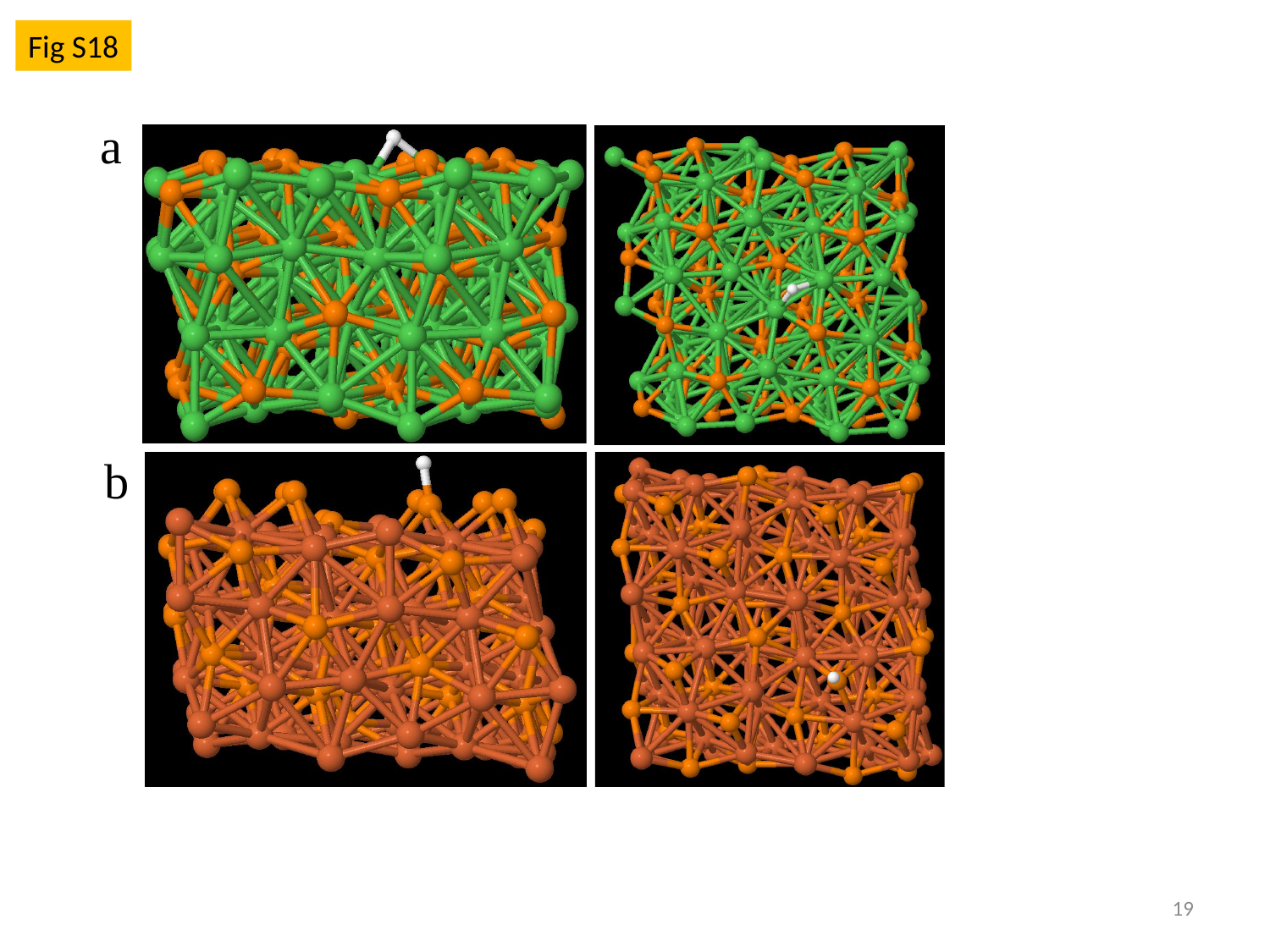

Fig S18
a
b
19

## Slide 20
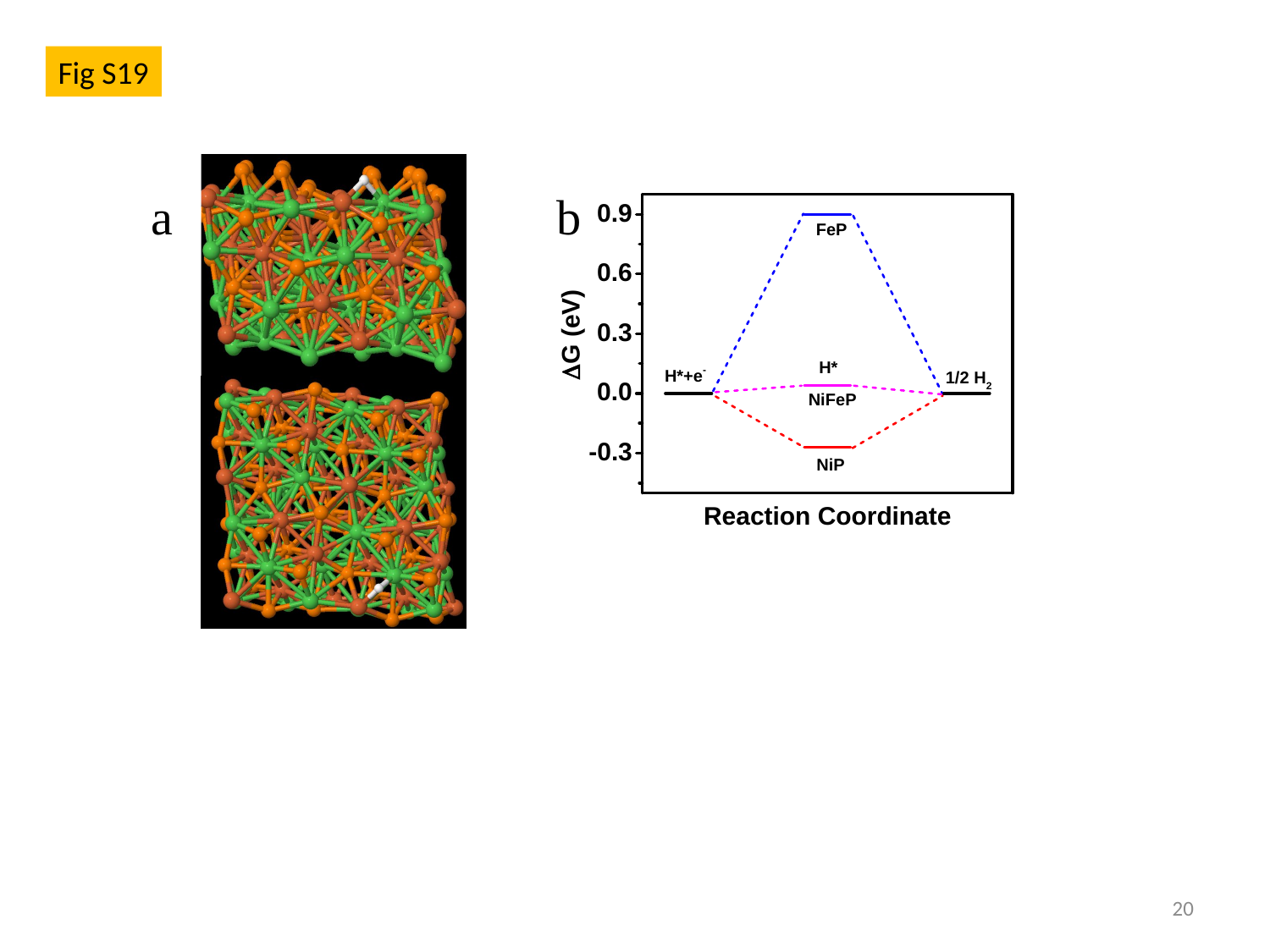

Fig S19
a
b
20

## Slide 21
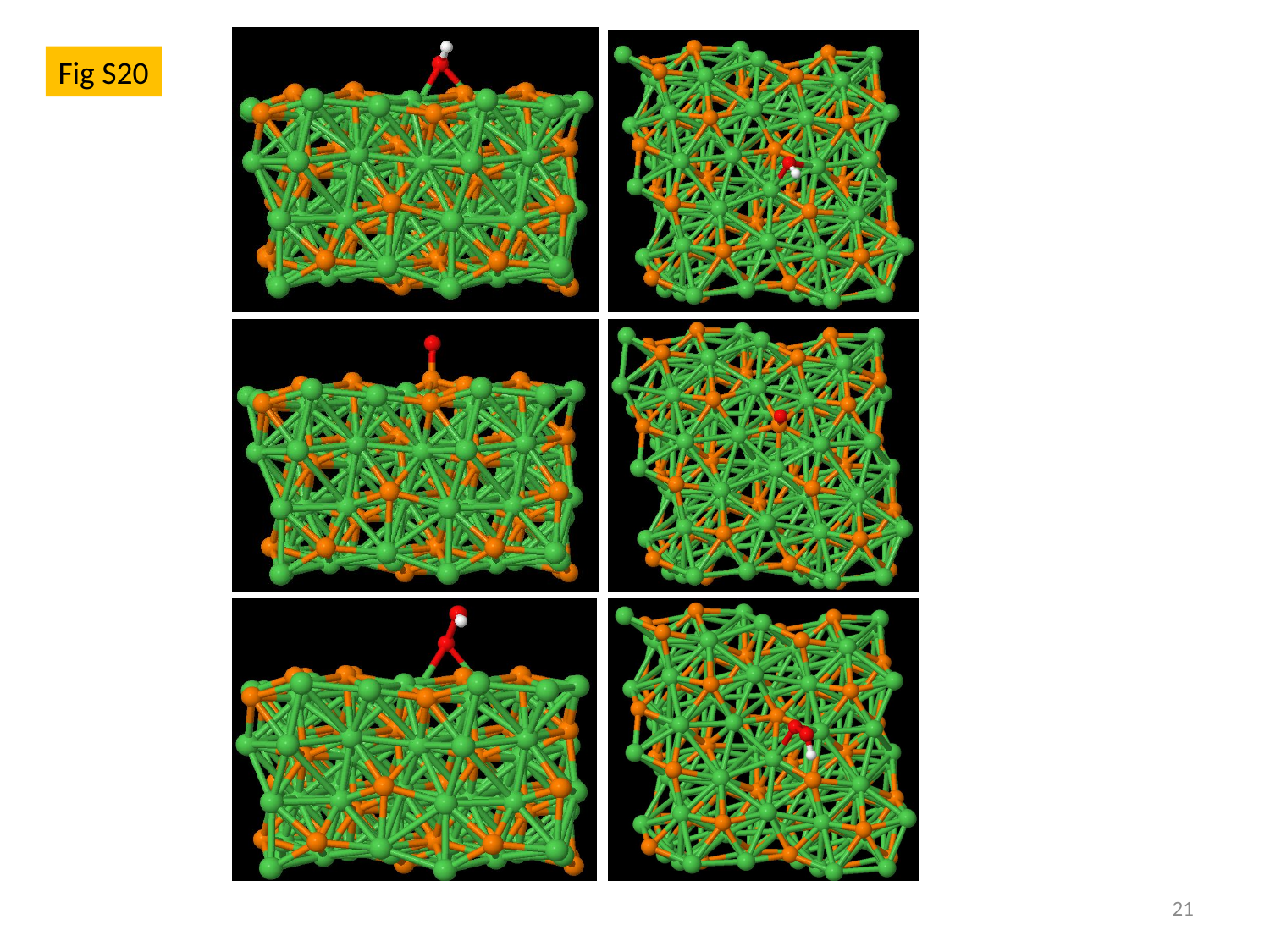

Fig S20
21

## Slide 22
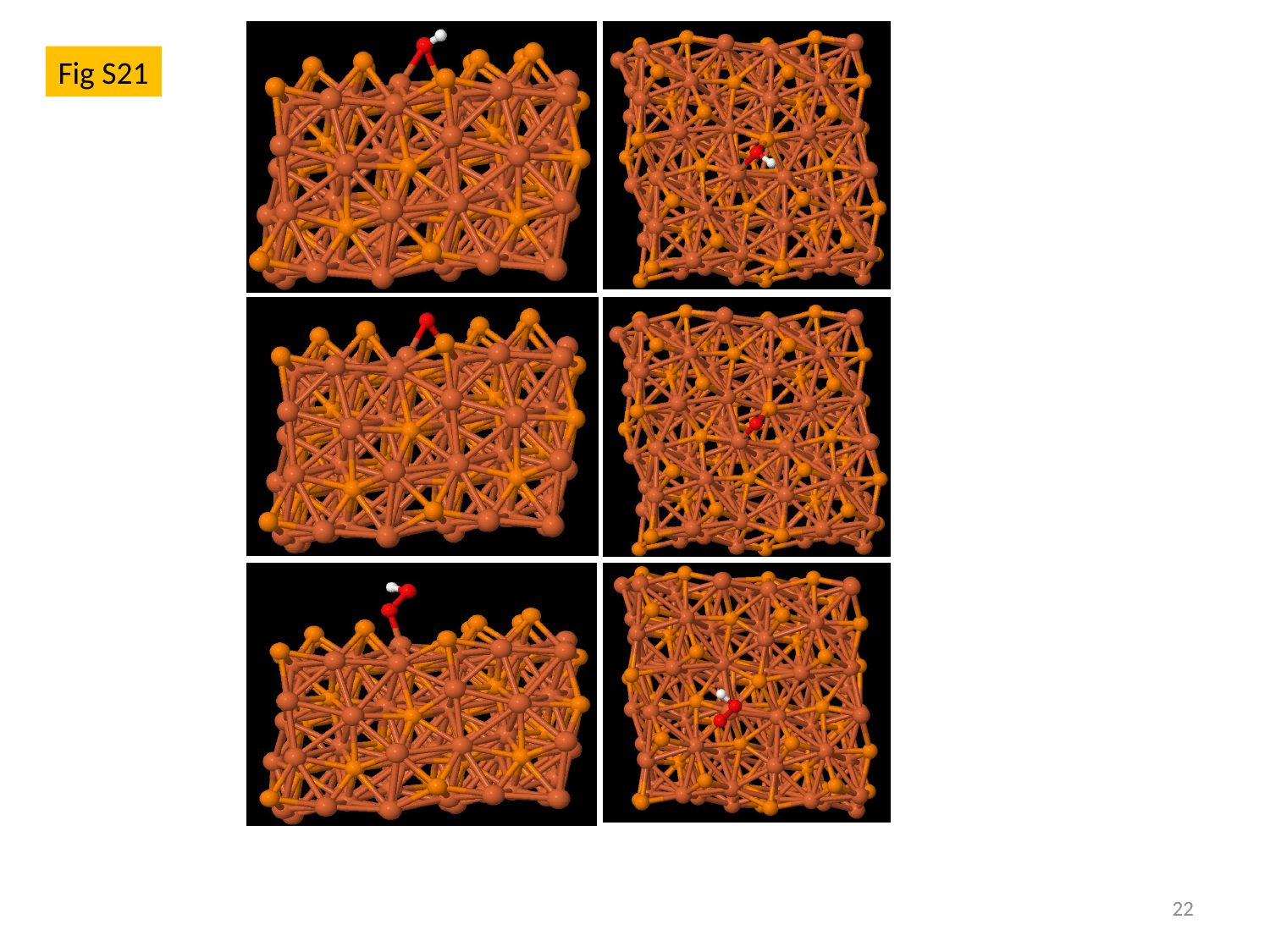

Fig S21
22

## Slide 23
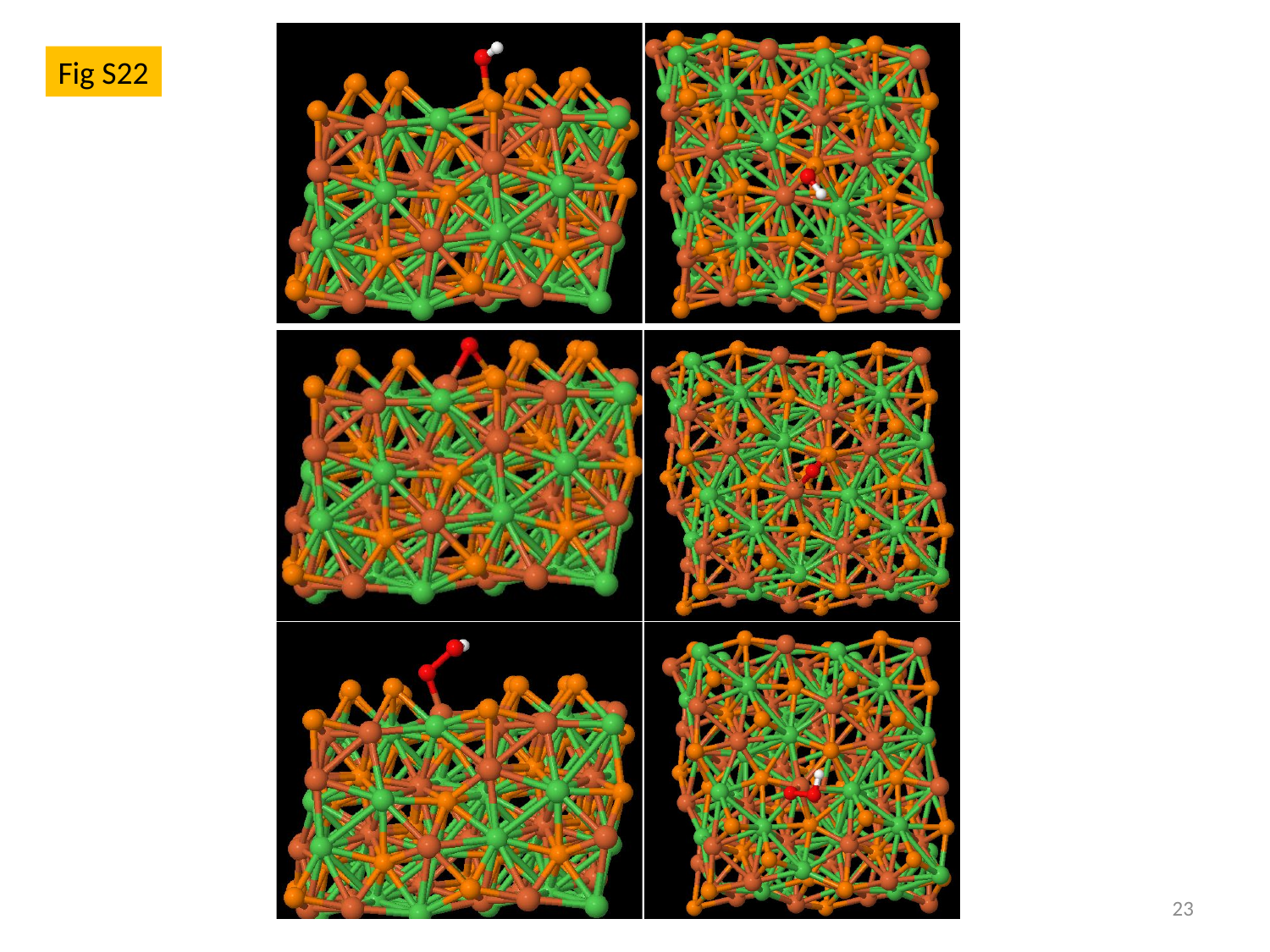

Fig S22
23

## Slide 24
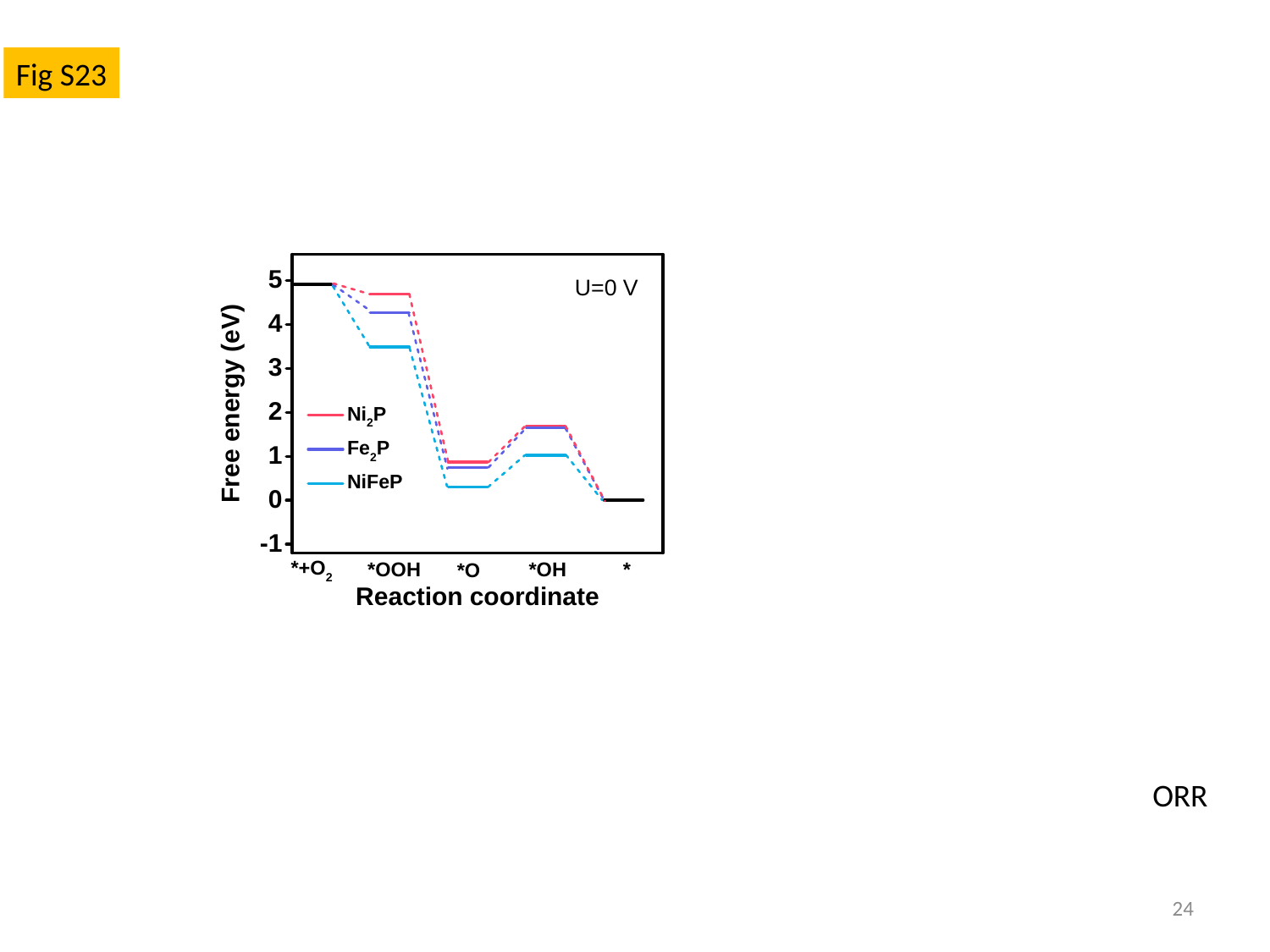

Fig S23
ORR
24

## Slide 25
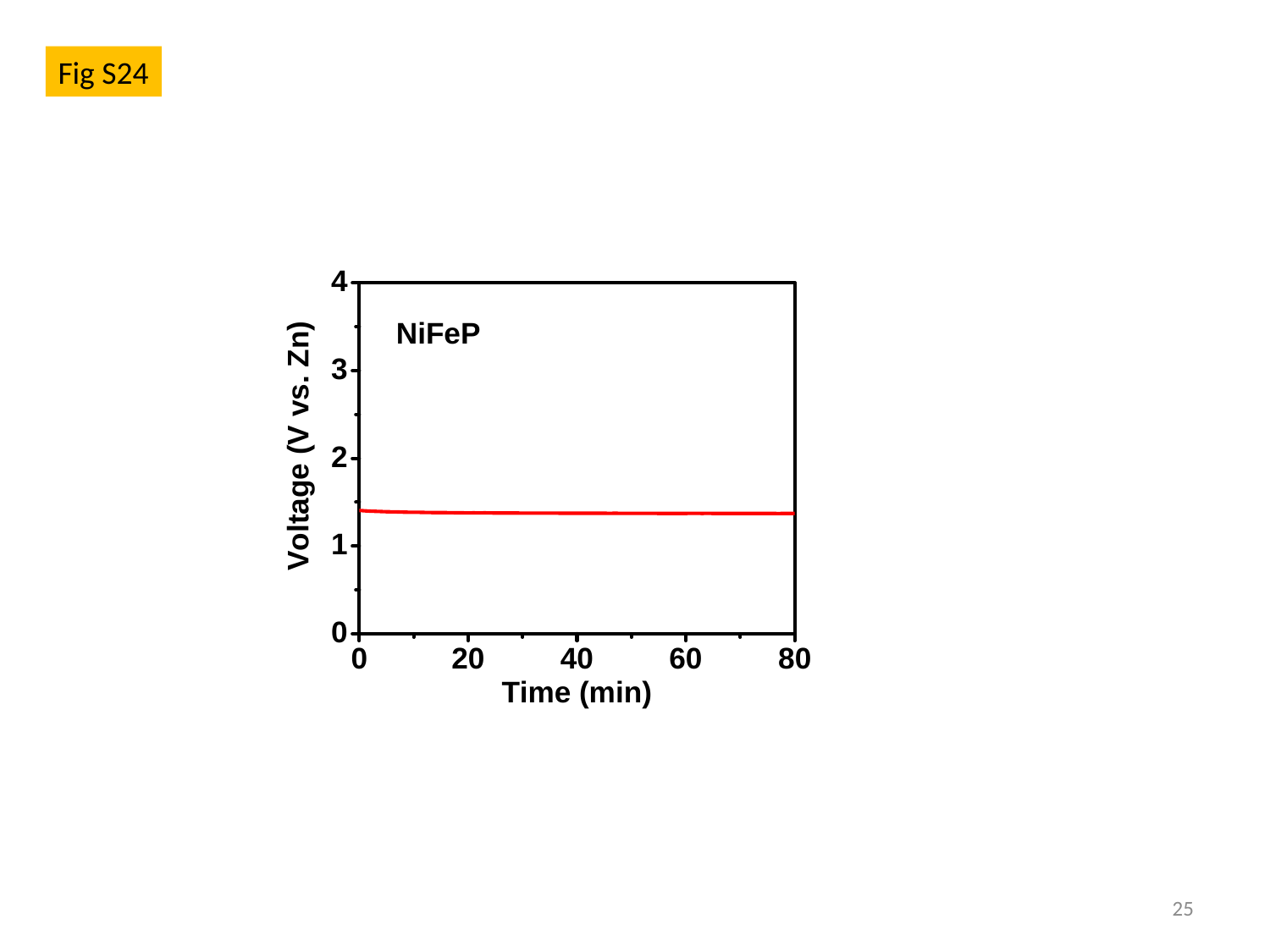

Fig S24
25

## Slide 26
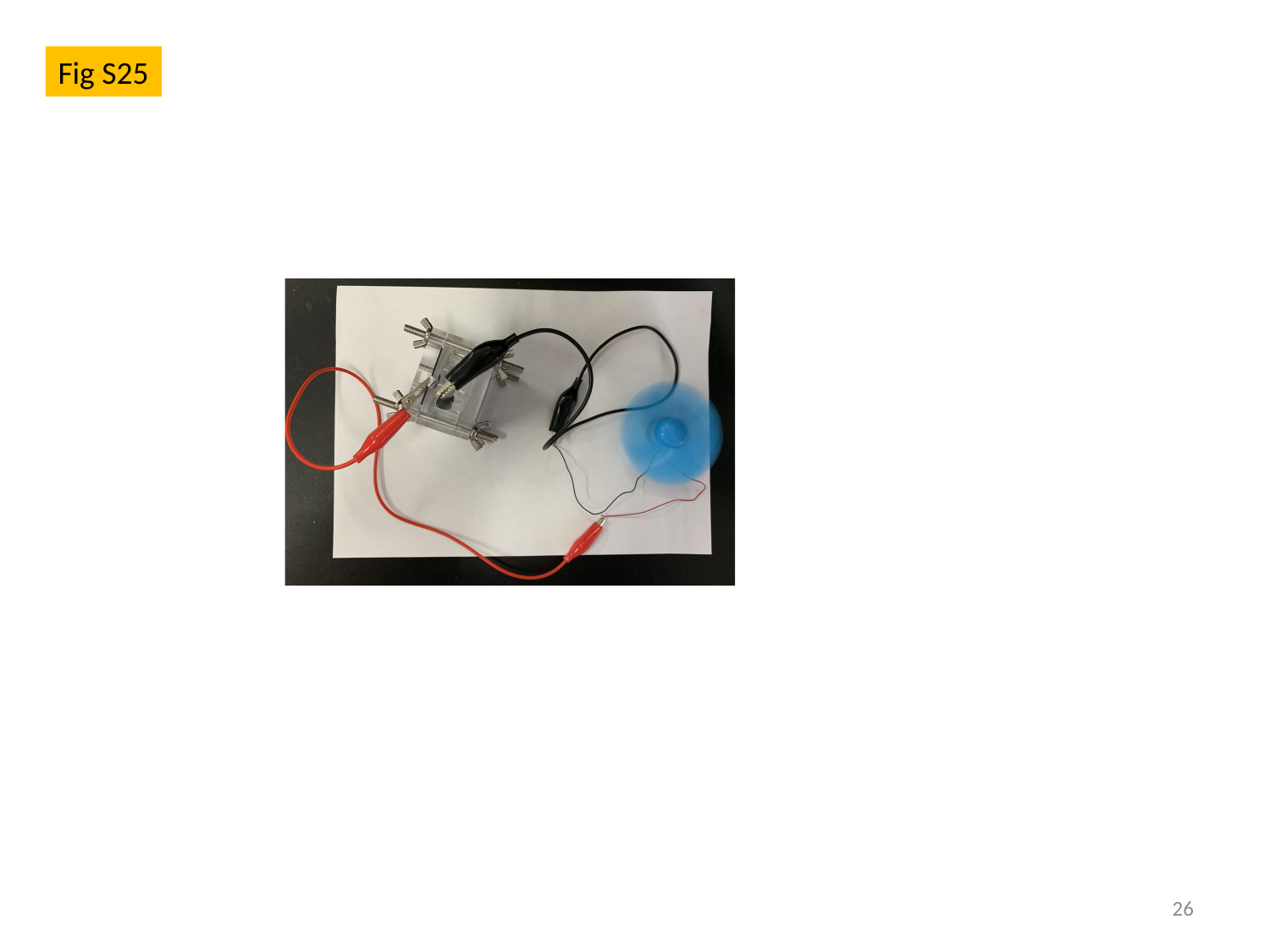

Fig S25
26

## Slide 27
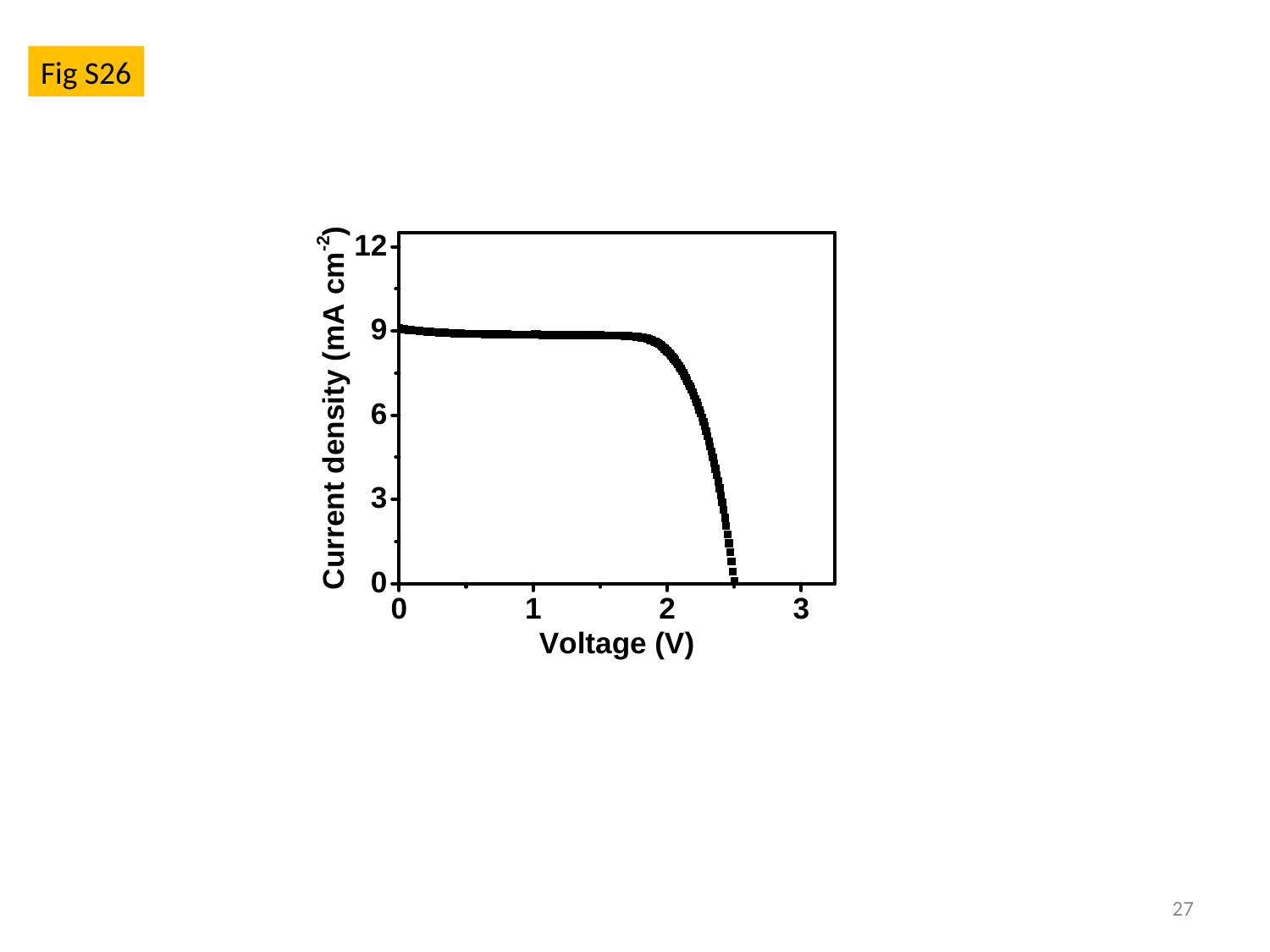

Fig S26
27

## Slide 28
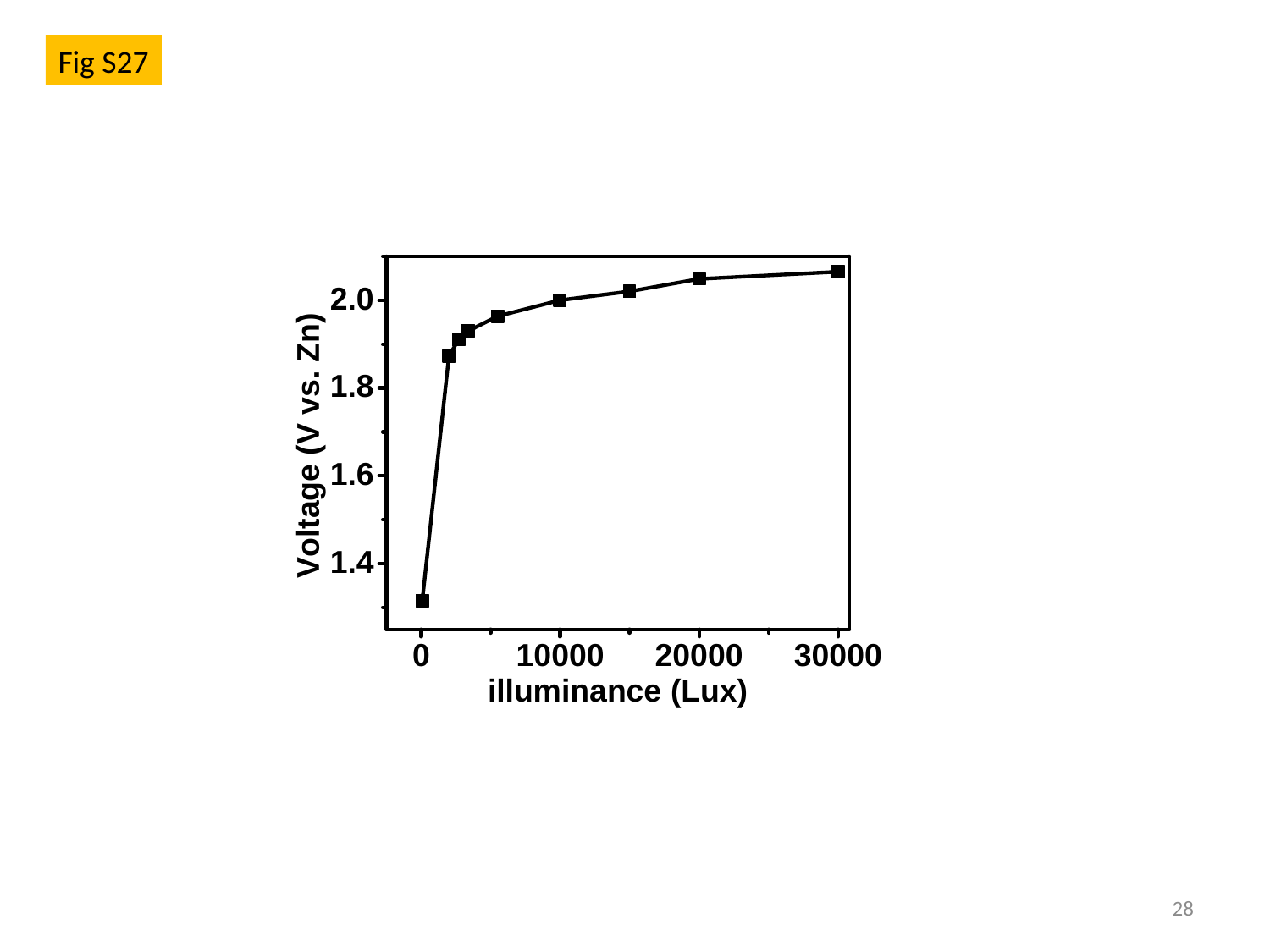

Fig S27
28

## Slide 29
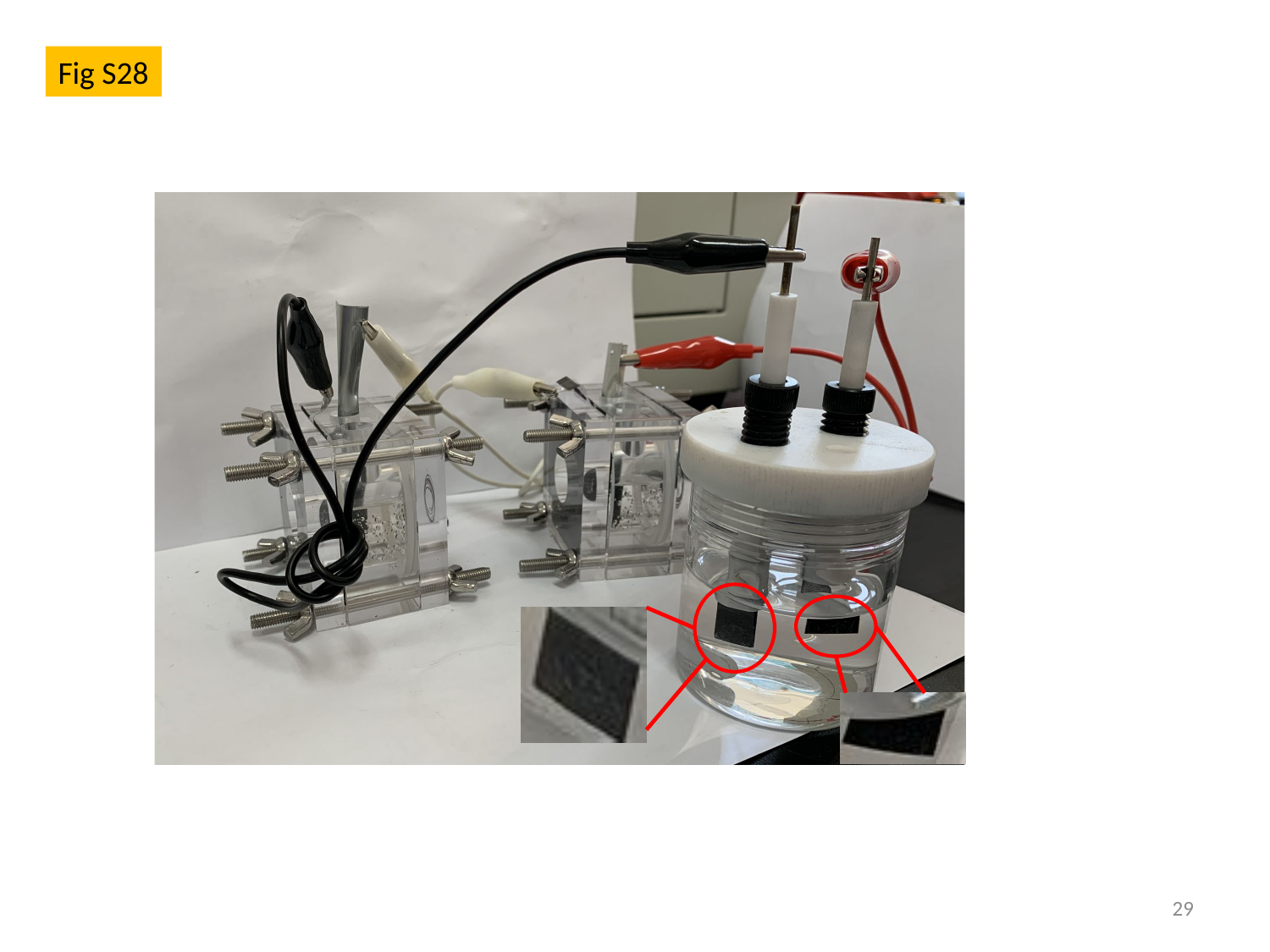

Fig S28
29

## Slide 30
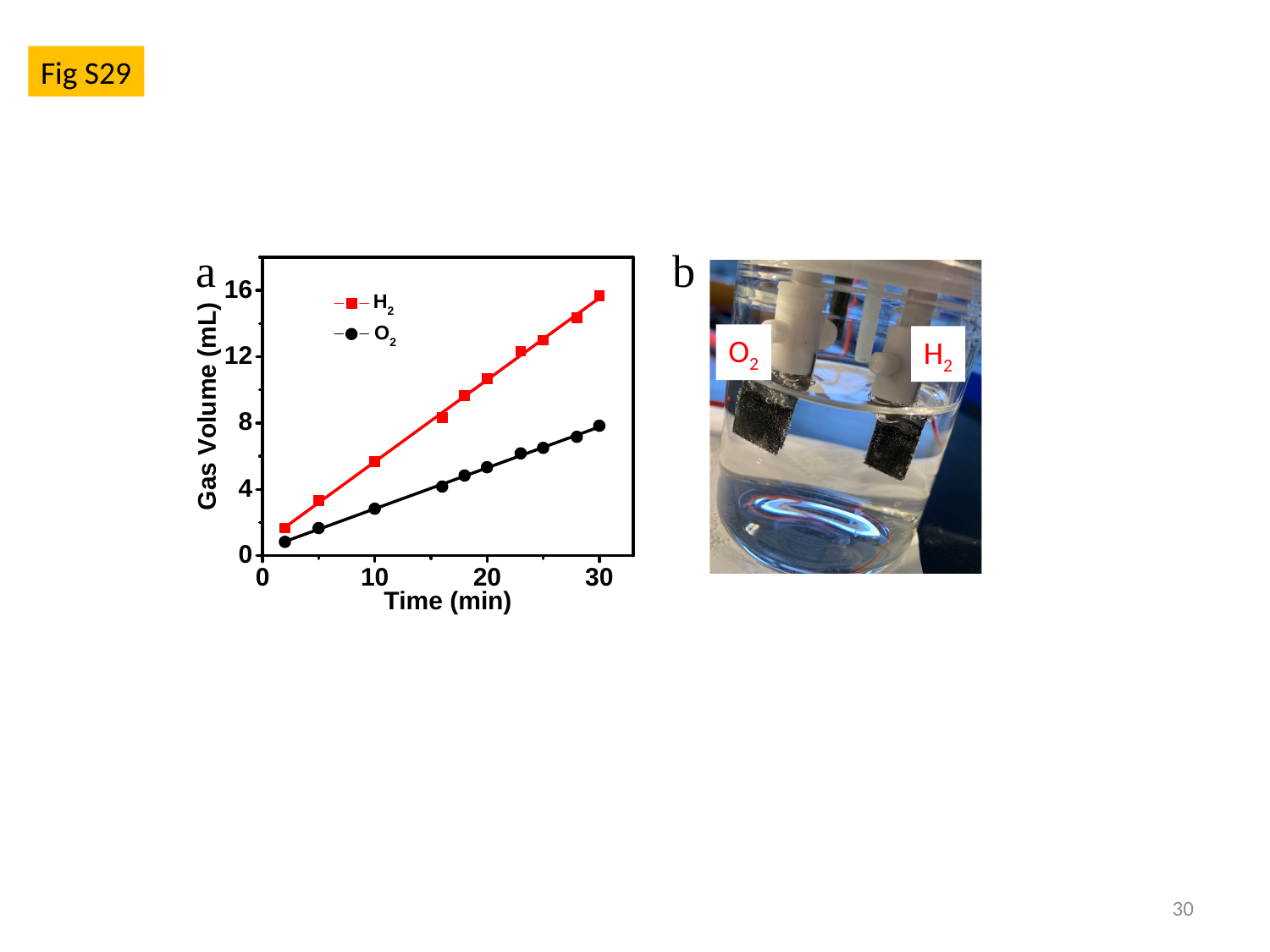

Fig S29
O2
H2
a
b
30
